# SlBBX19 modulates plant development and negatively regulates chilling tolerance in tomato

**DOI:** 10.1111/tpj.71099

**Published:** 2026-09-07

**Authors:** Yunfei Liang, Fanyi Meng, Hongwei Sun, Hongyan Shi, Fang Ma, Xiangqiang Zhan

**Affiliations:** ^1^ College of Grassland Agriculture Northwest A&F University Yangling 712100 China; ^2^ State Key Laboratory for Crop Stress Resistance and High‐Efficiency Production and College of Horticulture Northwest A&F University Yangling 712100 China

**Keywords:** tomato, chilling tolerance, BBX transcription factor, light‐harvesting chlorophyll a/b‐binding, photosynthesis, fruit ripening

## Abstract

Chilling stress severely restricts tomato cultivation and productivity, yet the mechanisms by which a single regulator integrates photosynthetic efficiency, developmental progression, and chilling tolerance remain to be elucidated. Here, we characterized *SlBBX19*, a chilling‐inducible B‐box transcription factor in tomato (*Solanum lycopersicum*). SlBBX19 is nucleus‐localized, functions as a negative regulator of vegetative growth and photosynthetic capacity under normal conditions, while simultaneously promoting fruit ripening and suppressing inflorescence branching. Under chilling stress, *SlBBX19* acts as a negative regulator of chilling tolerance: overexpression exacerbates reactive oxygen species (ROS) accumulation, membrane damage, and suppresses *COR* pathway activation, whereas knockout lines exhibit enhanced chilling resistance. Transcriptomic and molecular analyses reveal that SlBBX19 directly binds to G‐box elements in the promoters of light‐harvesting chlorophyll a/b‐binding genes *SlLhcb2.2* and *SlLhca4.1* to repress their transcription. Virus‐induced gene silencing of these targets recapitulated the overexpression phenotypes, confirming that *SlBBX19* compromises photosynthetic efficiency and chilling tolerance primarily through *SlLhc* downregulation. Collectively, our findings establish *SlBBX19* as a key integrator of development and environmental acclimation, revealing a novel regulatory module that links photosynthetic antenna dynamics to chilling stress adaptation. This work may provide a promising genetic target for engineering chilling‐tolerant and physiologically optimized tomato varieties.

## Significance Statement

Chilling stress severely limits tomato production, yet how plant development and chilling tolerance are coordinately regulated remains poorly understood. We identify the B‐box transcription factor SlBBX19 as a key regulator linking photosynthetic antenna gene expression with plant development and chilling responses, providing a promising genetic target for improving chilling tolerance and productivity in tomato.

## INTRODUCTION

In temperate agricultural regions worldwide, low temperature has emerged as a pivotal abiotic stress factor limiting normal crop development and compromising yield and quality (Ding et al., 2019; Zhu, 2016). Particularly during periods of drastic temperature fluctuations, such as early spring or late autumn, crops frequently sustain physiological damage upon exposure to non‐lethal low temperatures, which subsequently impairs photosynthetic efficiency and diminishes carbon assimilation capacity. Concurrently, dysfunction in the intracellular reactive oxygen species (ROS) scavenging system induces oxidative stress, resulting in lipid peroxidation of biological membranes and compromising cellular structural integrity (Gong et al., 2020). Tomato, a chilling‐sensitive crop widely cultivated in protected agriculture, exemplifies this vulnerability. Chilling injury in tomato not only inhibits flower bud differentiation and fruit setting but also precipitates delayed fruit development and an increased incidence of malformation, severely undermining market competitiveness. Consequently, improving tomato cold tolerance has become a priority for innovations in breeding and cultivation technologies. It is imperative to comprehensively elucidate the mechanisms underlying cold tolerance from multidimensional perspectives, including genetic regulation, metabolic pathways, and environmental management, to facilitate the breeding and dissemination of cold‐tolerant tomato varieties (Chong et al., 2025).

Low‐temperature stress significantly inhibits plant photosynthesis, rendering the photosynthetic apparatus particularly sensitive to cold environments (Xu, Zhang, et al., 2023, Li, Zhu, et al., 2024). Under chilling conditions, diminished light energy utilization and transfer efficiency often precipitate photoinhibition and excessive accumulation of ROS, thereby compromising the stability of the photosynthetic system (Xu, Wang, et al., 2023). The functionality of Photosystem II (PSII) depends not only on its reaction center proteins but also critically on the precise regulation of light capture and distribution by light‐harvesting chlorophyll a/b‐binding (Lhc) proteins (Shan et al., 2024). Numerous studies have indicated that multiple members of the *Lhc* families exhibit significant expression changes under low‐temperature stress. For example, *AcLhcb1.1* in kiwifruit is significantly upregulated following cold treatment, and overexpression of *AcLhcb1.1* enhances cold tolerance in kiwifruit (Liu et al., 2025). In tomato, *SlLhcb1.11* expression is suppressed by cold treatment; silencing *SlLhcb1.11* compromises tomato cold tolerance (Meng et al., 2025). Despite these insights, current research on *Lhc* gene expression under cold stress has primarily focused on transcriptional responses, leaving the upstream regulatory mechanisms largely unexplored. In particular, the specific transcription factors mediating this process in crops such as tomato remain to be elucidated.

Among the transcription factor families involved in light signaling, BBX proteins have emerged as important regulators integrating environmental cues with plant developmental programs. Given their key roles in photomorphogenesis and light‐responsive transcription, BBX proteins are attractive candidates for regulating *Lhc* genes during cold stress. However, whether BBX transcription factors directly control *Lhc* gene expression to modulate chilling tolerance remains unknown. The BBX (B‐box) transcription factor family is highly conserved in plants, with members typically containing one or two B‐box domains, and some also harboring a CCT domain (Bu et al., 2021; Lira et al., 2026). As a critical class of transcription factors, BBX proteins are extensively involved in diverse developmental processes, including photomorphogenesis, plant height regulation, flowering time, and fruit development (Gao et al., 2026; Pan et al., 2025; Yang et al., 2024; Zhang et al., 2026). Recent studies have further revealed that BBX family members participate in responses to various abiotic stresses, such as low and high temperatures, drought, and salt‐alkali stress (Du et al., 2026; Li, Ai, et al., 2024; Song et al., 2023; Wang & Zhan, 2024; Wu et al., 2026). Notably, specific BBX proteins modulate plant cold tolerance by either positively or negatively regulating the expression of cold‐responsive genes. For instance, *AtBBX29* in Arabidopsis acts as a negative regulator of cold tolerance, with transcriptome analyses revealing its regulation of thousands of cold‐responsive (*COR*) genes (Wang, Shen, et al., 2023). In tomato, *SlBBX17* interacts with ELONGATED HYPOCOTYL5 (SlHY5) to enhance SlHY5 protein stability, thereby promoting the transcriptional activation of *SlCBFs* by *SlHY5* under cold stress (Song et al., 2023). Similarly, *SlBBX31* positively regulates cold tolerance by directly binding to and activating *C‐repeat binding factor* (*SlCBF*) expression (Zhu et al., 2023). Mechanistically, BBX proteins often function as a bridge between light signaling and environmental stress responses by regulating the transcription of light‐ or cold‐responsive genes or by interacting with key light signaling components such as HY5. These observations raise the possibility that BBX transcription factors contribute to chilling tolerance by regulating *Lhc* genes. However, whether BBX proteins directly regulate *Lhc* genes during cold stress has not yet been demonstrated in tomato.

In Arabidopsis, BBX19 has been well‐documented to participate in light signaling and developmental regulation. For instance, BBX19 interacts with CONSTANS (CO) to repress the transcription of *FLOWERING LOCUS T* (*FT*), thereby delaying flowering time (Wang et al., 2014). As a key regulator of light signaling, *BBX19* promotes hypocotyl elongation and influences photomorphogenesis; this function relies on CONSTITUTIVE PHOTOMORPHOGENIC1 (COP1)‐mediated degradation of EARLY FLOWERING3 (ELF3) (Wang et al., 2015), highlighting the critical role of BBX19 in photomorphogenesis. Furthermore, BBX19 interacts with PSEUDO‐RESPONSE REGULATORS (PRR9/7/5) to modulate the expression of circadian clock genes, integrating into the circadian regulatory network and underscoring its complex role in light environmental responses (Yuan et al., 2021). However, the function of SlBBX19 in tomato remains largely uncharacterized. Specifically, its role in cold stress response and photosynthetic system regulation has not been systematically investigated. Therefore, we hypothesized that SlBBX19 functions as a molecular link between light signaling and chilling adaptation by regulating *Lhc* genes.

Here, we aim to elucidate the function of *SlBBX19* in tomato development and cold stress response. By generating *SlBBX19* overexpression and knockout lines, we systematically analyzed its roles in plant development, photosynthesis, and cold tolerance. Our results demonstrate that *SlBBX19* overexpression leads to dwarfism, leaf chlorosis, reduced flower number, and accelerated fruit maturation, whereas knockout plants exhibit the opposite phenotypes. Notably, *SlBBX19*‐overexpressing plants display increased sensitivity to low temperature, while knockout plants exhibit enhanced cold tolerance. Mechanistically, *SlBBX19* directly binds to and represses the transcription of *SlLhcb2.2* and *SlLhca4.1*, thereby reducing photosynthetic efficiency. This study uncovers a regulatory module linking light harvesting, cold tolerance, and developmental processes, providing new theoretical insights into the crosstalk between light signaling and stress responses, and offering potential genetic targets for breeding chilling‐tolerant tomato varieties.

## RESULTS

### *SlBBX19* is a chilling‐responsive BBX transcription factor

**Figure 1:**
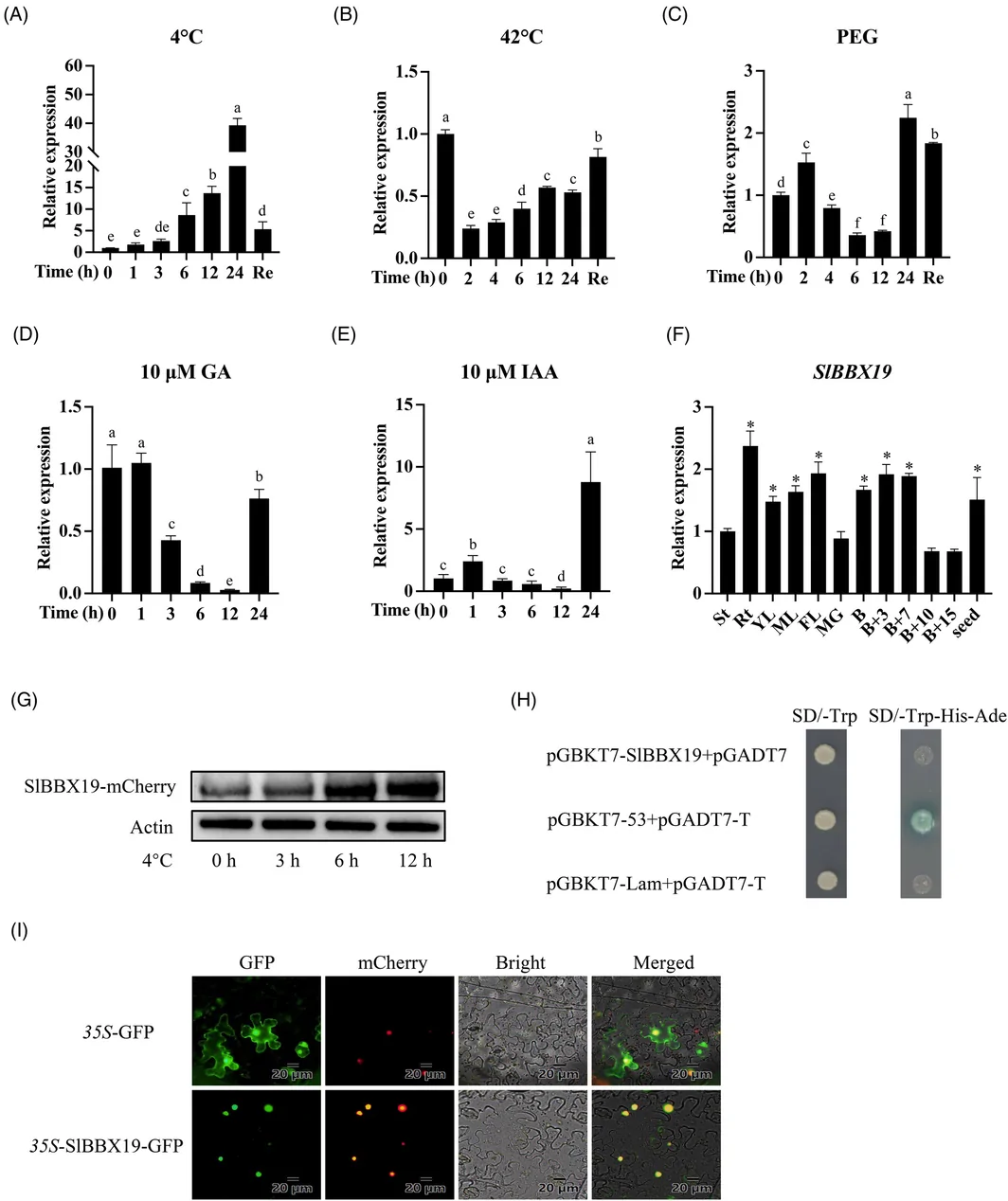
Expression pattern of *SlBBX19* and subcellular localization of SlBBX19. (A–E) Relative expression of *SlBBX19* in tomato seedlings following exposure to chilling (4°C), high temperature (42°C), drought stress simulated by 15% PEG, or exogenous hormones (10 μM GA or 10 μM IAA). Error bars represent SD values. The letters indicate significant differences according to one‐way ANOVA (Tukey's test; *P* < 0.05). (F) Tissue‐specific and developmental expression profile of *SlBBX19* across stem (ST), root (RT), young leaves (YL), mature leaves (ML), flowers (FL), mature green fruit (MG), breaker fruit (Br), fruit harvested 3–15 days post‐breaker (Br + 3–15), and seeds. Error bars represent SD values. Statistical analysis was performed by two‐tailed Student's *t*‐test (*P < 0.05). (G) Chilling‐induced accumulation of SlBBX19 protein in transiently transformed *Nicotiana benthamiana* leaves. Leaves expressing SlBBX19‐mCherry were subjected to chilling treatment for 0, 3, 6, and 12 h. Total proteins were detected using an anti‐mCherry antibody. (H) Yeast one‐hybrid assay for transcriptional activation activity of SlBBX19. Co‐transformation of pGBKT7‐53 and pGADT7‐T was used as a positive control, while pGBKT7‐Lam and pGADT7‐T served as a negative control. (I) Subcellular localization of SlBBX19. SlBBX19‐GFP fusion protein was transiently expressed in *N. benthamiana* leaves, with empty GFP serving as a control. mCherry signals indicate a nuclear localization marker. Scale bars = 20 μm.

To identify key regulatory factors involved in the chilling response in tomato, we analyzed RNA‐seq data generated from our previous chilling stress (accession numbers: SAMN14996375‐14996413). To obtain a comprehensive overview of the response of the tomato *BBX* family to chilling stress, the expression patterns of all 31 *BBX* genes were analyzed under chilling treatment. Several members, including *SlBBX7*, *SlBBX17*, *SlBBX19*, *SlBBX22*, *SlBBX28*, and *SlBBX31*, were markedly upregulated, whereas *SlBBX4*, *SlBBX8*, *SlBBX13*, *SlBBX14*, and *SlBBX26* were progressively downregulated (Figure S1). Among these cold‐induced members, *SlBBX19* was selected for further functional characterization because its biological function in tomato chilling tolerance remains largely unknown. To further validate this finding and characterize its expression dynamics, we performed quantitative real‐time PCR (qRT‐PCR) to analyze the expression profile of *SlBBX19* under various abiotic stresses. *SlBBX19* exhibited a robust response to 4°C treatment; its transcript levels accumulated significantly over time during 4°C treatment, peaking at 24 h with an approximately 40‐fold increase compared to the control (Figure 1A). In contrast, *SlBBX19* expression was generally suppressed under heat stress (Figure 1B). Under PEG‐simulated drought stress, *SlBBX19* displayed a dynamic expression pattern: it showed a transient increase at the early stage, dropped to a minimum at 6 h, and subsequently rebounded to reach its highest level at 24 h. Furthermore, plant hormone treatments significantly modulated *SlBBX19* transcription. Gibberellin (GA) treatment resulted in significant mid‐stage repression followed by recovery in the late stage, whereas auxin (IAA) treatment induced a delayed but strong upregulation, with expression levels rising significantly after 24 h (Figure 1D,E). Tissue‐specific expression analysis revealed that *SlBBX19* was highly expressed in roots and flowers, whereas its expression levels were relatively low in green mature (MG) and red ripe (RR) fruits (Figure 1F). We also examined SlBBX19 protein accumulation under chilling stress using transiently expressed SlBBX19‐mCherry in *Nicotiana benthamiana* leaves. Immunoblot analysis showed that SlBBX19 protein abundance gradually increased after chilling treatment, indicating that BBX19 protein is induced by low temperature in addition to the previously observed transcriptional induction (Figure 1G). Phylogenetic analysis revealed that *SlBBX19* clusters closely with *SlBBX18* within a specific subclade of the tomato BBX family (Figure S2). Notably, *SlBBX18* has been reported to negatively regulate tomato drought tolerance (Li, Ai, et al., 2024). Additionally, yeast self‐activation assays indicated that *SlBBX19* lacks intrinsic transcriptional self‐activation activity (Figure 1H). To investigate the subcellular localization of *SlBBX19*, we constructed a *SlBBX19*‐GFP fusion protein and transiently expressed it in *N. benthamiana* leaves. Microscopy revealed that the fusion protein signal was predominantly localized in the nucleus, consistent with its predicted function as a transcription factor (Figure 1I). Collectively, these results demonstrate that *SlBBX19* is a chilling‐inducible, nucleus‐localized transcription factor with specific tissue expression patterns.

### *SlBBX19* negatively regulates plant vegetative growth and photosynthesis

**Figure 2:**
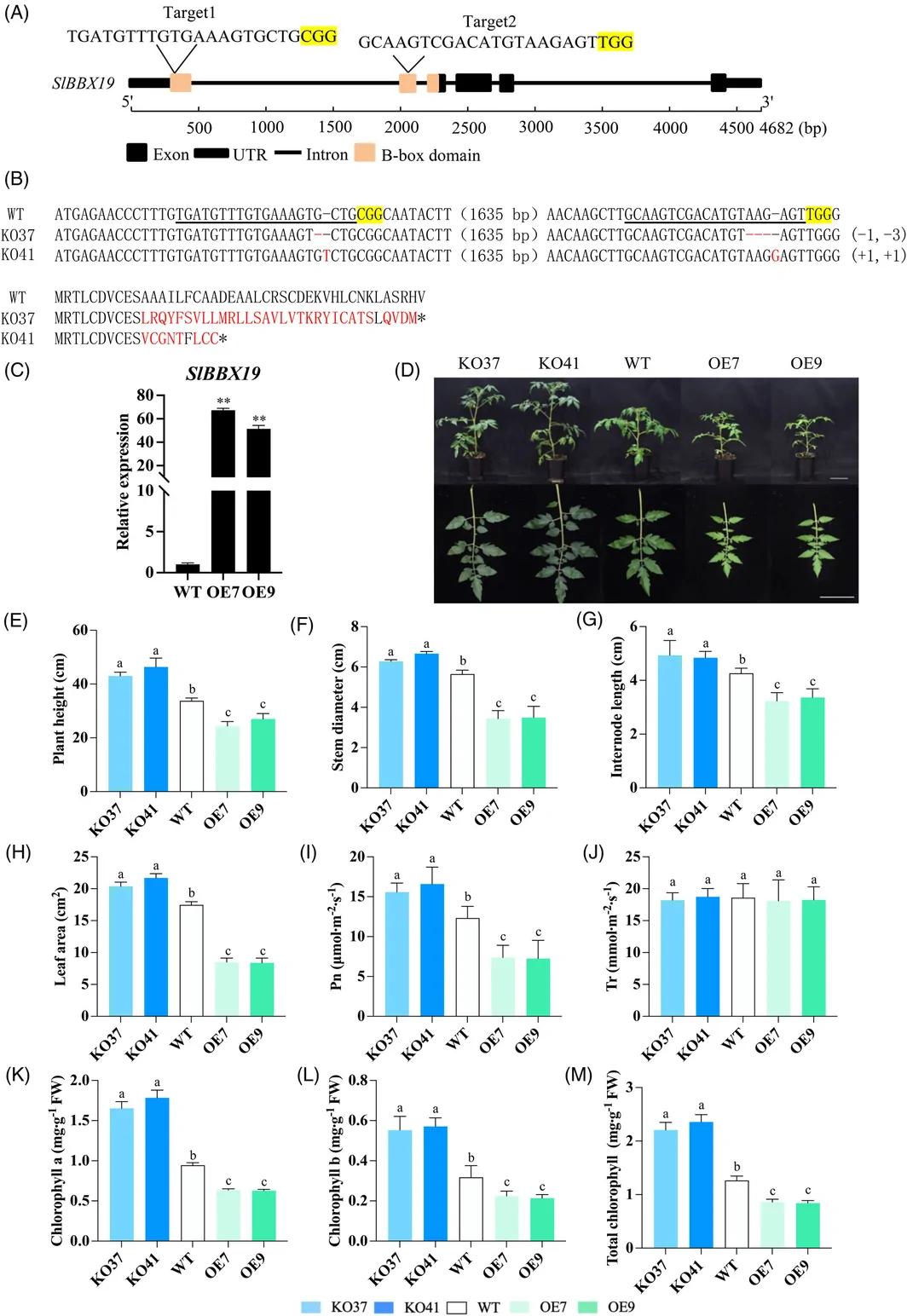
*SlBBX19* negatively regulates vegetative growth in tomato. (A) Schematic diagram of the *SlBBX19* gene structure and genome‐editing target sites. (B) Genomic DNA sequence alignment at the target locus and corresponding predicted amino acid alterations in *SlBBX19* knockout (KO) lines. (C) Quantitative RT‐PCR analysis of *SlBBX19* transcript levels in overexpression lines relative to WT. Error bars represent SD values. Statistical analysis was performed by two‐tailed Student's *t*‐test (**P < 0.01). (D) Representative photographs of vegetative phenotypes in WT, *SlBBX19*‐KO, and *SlBBX19*‐OE plants at 70 days after seeding. Scale bars = 10 cm. (E–J) Quantitative measurements of morphological and physiological parameters: (E) plant height, (F) stem diameter, (G) internode length, (H) leaf area, (I) net photosynthetic rate (Pn), and (J) transpiration rate (Tr). (K–M) Chlorophyll content in leaves: (K) chlorophyll a, (L) chlorophyll b, and (M) total chlorophyll. In panel E–M, error bars represent SD values. The letters indicate significant differences according to one‐way ANOVA (Tukey's test; *P* < 0.05).

To elucidate the biological function of *SlBBX19* in tomato vegetative growth, we generated *SlBBX19* knockout (*SlBBX19*‐KO) and overexpression (*SlBBX19*‐OE) transgenic lines using *Agrobacterium*‐mediated transformation. Molecular characterization confirmed the successful generation of these lines: in two independent knockout lines (KO37 and KO41), frameshift mutations were detected in the *SlBBX19* coding sequence, leading to premature translation termination (Figure 2A,B). Conversely, in two overexpression lines (OE7 and OE9), the transcript levels of *SlBBX19* were approximately 67‐fold and 51‐fold higher, respectively, compared to the wild type (WT) (Figure 2C). After 70 days of growth under normal conditions, distinct morphological phenotypes were observed among the genotypes. The *SlBBX19*‐KO plants exhibited an overgrowth phenotype, with significantly increased plant height, stem diameter, internode length, and leaf area compared to WT plants. In contrast, the *SlBBX19*‐OE plants displayed a dwarf phenotype, characterized by significantly reduced plant height, stem diameter, internode length, and leaf area (Figure 2E–H). Physiological assays further revealed the impact of *SlBBX19* on the photosynthetic apparatus. The *SlBBX19*‐KO plants showed significantly higher chlorophyll content and net photosynthetic rate than WT plants. Conversely, *SlBBX19*‐OE plants exhibited reduced chlorophyll content and a lower net photosynthetic rate (Figure 2I–M). Notably, despite the significant differences in net photosynthetic rates among genotypes, the transpiration rate (Tr) remained statistically unchanged across all lines (Figure 2J). This suggests that the *SlBBX19*‐mediated regulation of photosynthesis is likely driven by non‐stomatal limitations, such as alterations in the photosynthetic machinery, rather than changes in stomatal conductance. Collectively, these findings identify *SlBBX19* as a key negative regulator coordinating vegetative growth and photosynthetic efficiency.

### *SlBBX19* inhibits inflorescence branching and promotes fruit ripening

**Figure 3:**
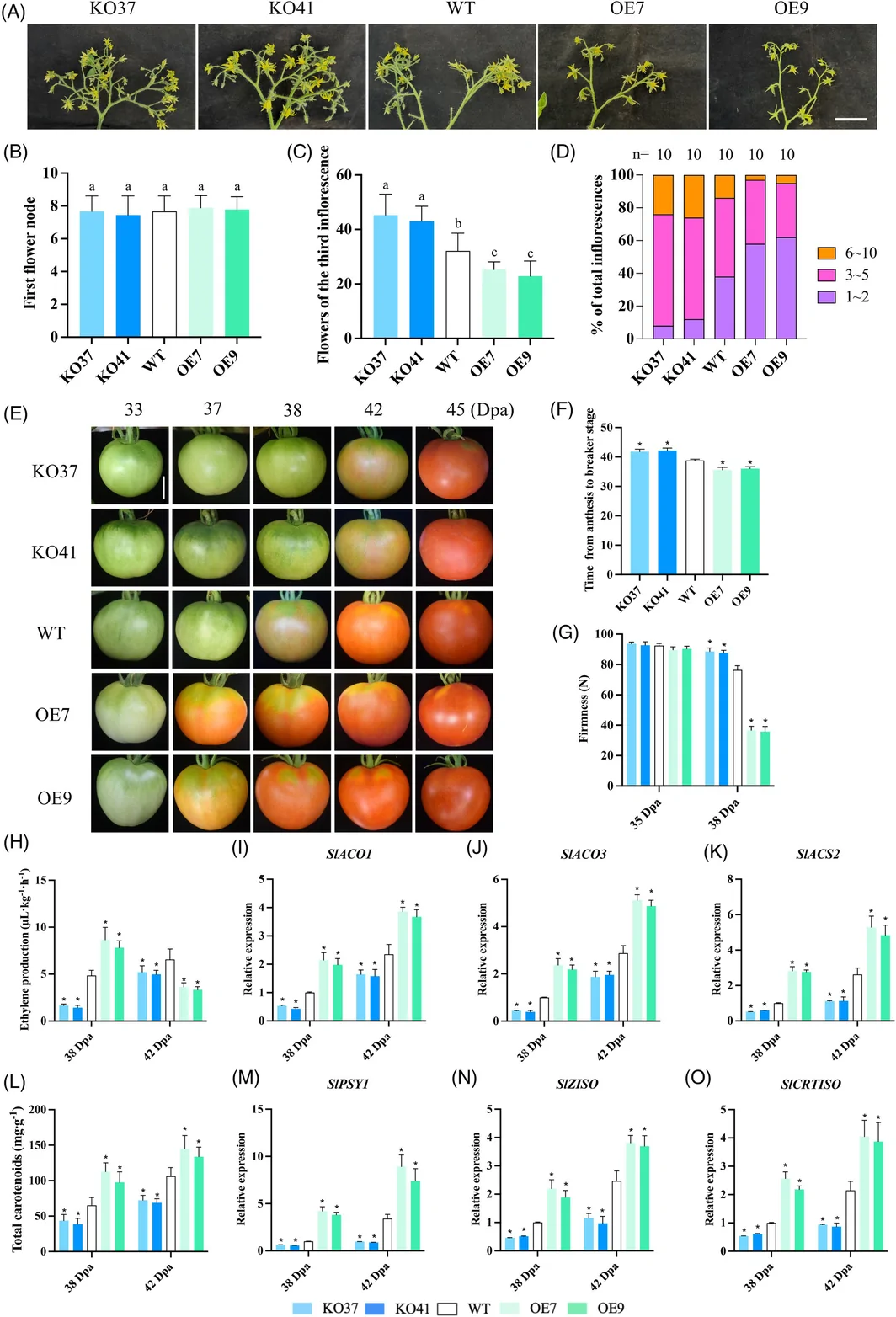
*SlBBX19* suppresses inflorescence branching and accelerates fruit ripening. (A) Representative inflorescence photos of WT, *SlBBX19*‐KO, and *SlBBX19*‐OE plants. Scale bar = 5 cm. (B) Node position of the first inflorescence. Error bars represent SD values. The letters indicate significant differences according to one‐way ANOVA (Tukey's test; *P* < 0.05). (C) Number of flowers on the third inflorescence. Error bars represent SD values. The letters indicate significant differences according to one‐way ANOVA (Tukey's test; *P* < 0.05). (D) Quantification of inflorescence branching in WT, *SlBBX19*‐KO, and *SlBBX19*‐OE plants. (E) Fruit ripening progression of WT, *SlBBX19*‐KO, and *SlBBX19*‐OE plants at 33‐, 37‐, 38‐, 42‐, and 45‐days post anthesis (Dpa). Scale bar = 1.5 cm. (F) Time from anthesis to breaker stage in different genotypes. (G) Fruit firmness at 35 and 38 Dpa in different genotypes. (H) Endogenous ethylene emission rates during fruit development in different genotypes. (I–K) Relative expression levels of ethylene biosynthesis genes in different genotypes. (L) Total carotenoid content in WT, *SlBBX19*‐KO, and *SlBBX19*‐OE fruits. (M–O) Relative expression levels of carotenoid biosynthesis genes in different genotypes. In panel F–O, error bars represent SD values. Statistical analysis was performed by two‐tailed Student's *t*‐test (*P < 0.05).

To investigate the impact of *SlBBX19* on reproductive development, we first analyzed inflorescence morphology. While the first‐flower node position showed no significant differences among genotypes (Figure 3B), the branching capacity of inflorescences varied markedly. In the third inflorescence, *SlBBX19*‐KO plants produced significantly more flowers than WT, whereas *SlBBX19*‐OE plants produced the fewest (Figure 3C). Statistical analysis revealed that *SlBBX19* acts as a potent inhibitor of inflorescence branching: approximately 90% of inflorescences in *SlBBX19*‐KO plants possessed three or more branches, compared to 62% in WT and only 40% in OE lines. Furthermore, the proportion of inflorescences with six or more branches was ~25% in KO plants, versus 14% in WT and less than 5% in OE plants (Figure 3D). These results indicate that *SlBBX19* functions as a negative regulator of inflorescence branching. In contrast, *SlBBX19* positively regulates fruit ripening. *SlBBX19*‐KO plants exhibited a significant delay in fruit maturation, whereas *SlBBX19*‐OE plants displayed an accelerated ripening phenotype (Figure 3E). Physiological assays at 38 days post anthesis (Dpa) showed that ethylene production and carotenoid content were significantly higher in *SlBBX19*‐OE fruits compared to WT, while *SlBBX19*‐KO fruits had the lowest levels. By 42 Dpa, although ethylene production in *SlBBX19*‐OE fruits declined (with WT fruits reaching a peak at this stage), carotenoid accumulation continued to be highest in OE fruits, followed by WT, and lowest in KO fruits (Figure 3H,L). Molecular analysis further confirmed that *SlBBX19* positively regulates the expression of genes involved in fruit maturation. The transcript levels of key ethylene biosynthesis genes (*SlACO1*, *SlACO3*, *SlACS2*) and carotenoid biosynthesis genes (*SlPSY1*, *SlZISO*, *SlCRTISO*) were significantly upregulated in *SlBBX19*‐OE plants but suppressed in *SlBBX19*‐KO plants (Figure 3I–K,M–O). Although *SlBBX19* promoted fruit maturation, no significant differences were observed in single fruit weight or fruit size between *SlBBX19*‐OE, *SlBBX19*‐KO, and WT plants (Figure S3). Collectively, these findings demonstrate that *SlBBX19* promotes fruit ripening by activating ethylene and carotenoid biosynthesis pathways.

### *SlBBX19* negatively regulates chilling tolerance

**Figure 4:**
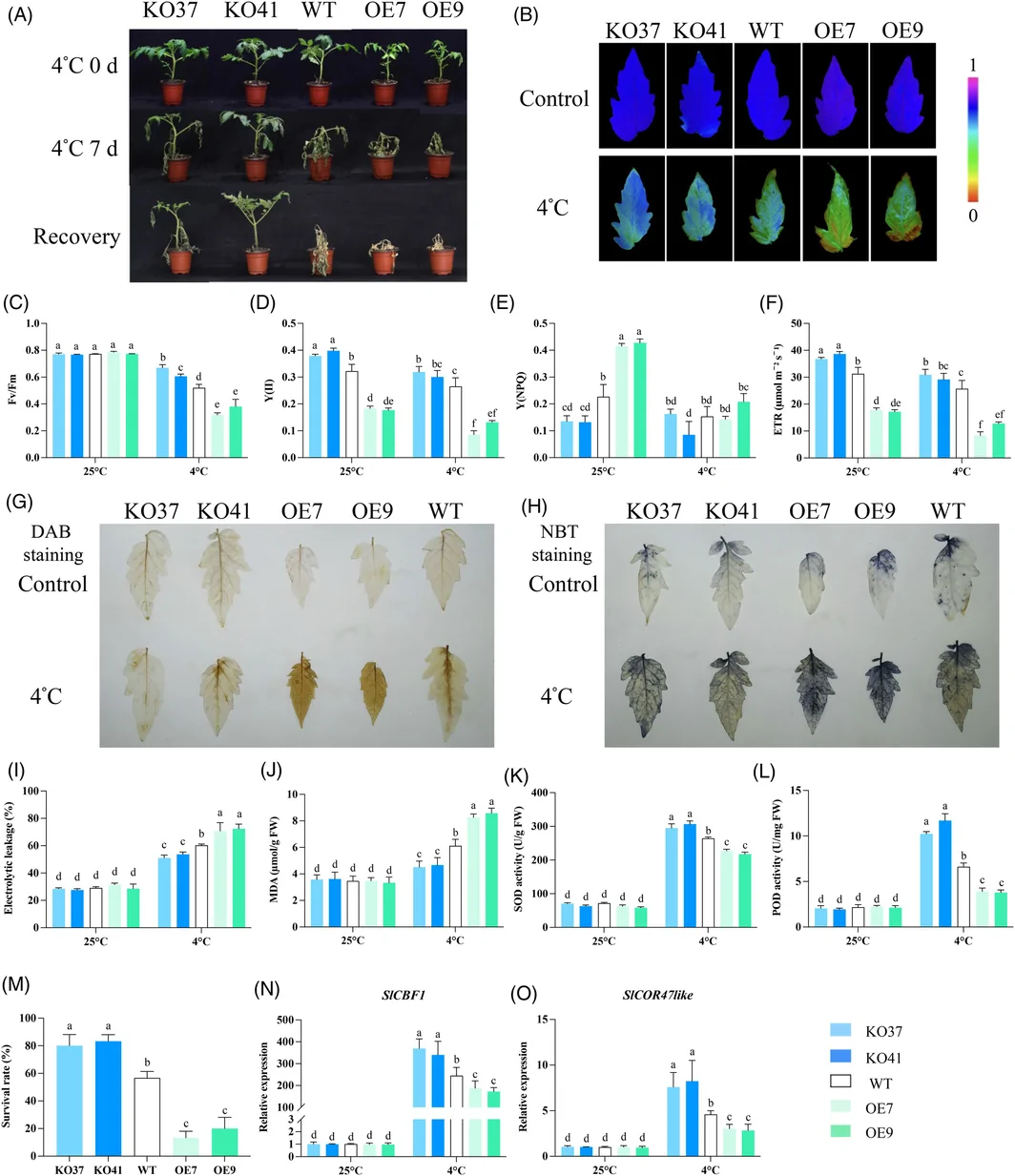
*SlBBX19* negatively regulates chilling tolerance in tomato. (A) Phenotypes of WT, *SlBBX19*‐KO, and *SlBBX19*‐OE seedlings following chilling treatment and recovery. Seedlings were subjected to chilling treatment at 4°C for 7 days, followed by recovery under normal growth conditions for 2 days. Representative images are shown. For survival analysis, eight plants per genotype were evaluated in each independent experiment, and three independent experiments were performed. (B–F) Chlorophyll fluorescence parameters of WT, *SlBBX19*‐KO, and *SlBBX19*‐OE seedlings under normal and chilling stress: (B, C) Maximum quantum efficiency of PSII (Fv/Fm), (D) Effective quantum yield of PSII (Y(II)), (E) Quantum yield of regulated non‐photochemical energy dissipation (Y(NPQ)), (F) Electron transport rate (ETR). (G, H) DAB staining and NBT staining for reactive oxygen species (ROS) accumulation in tomato leaves under control and chilling conditions. (I) Relative electrolytic leakage in WT, *SlBBX19*‐KO, and *SlBBX19*‐OE seedlings under normal and chilling stress. (J) Malondialdehyde (MDA) content in WT, *SlBBX19*‐KO, and *SlBBX19*‐OE seedlings under normal and chilling stress. (K, L) Activities of antioxidant enzymes: (K) superoxide dismutase (SOD) and (L) peroxidase (POD). (M) Plant survival rate after recovery from chilling stress. (N, O) Transcript levels of cold‐responsive genes (*SlCBF1* and *SlCOR47*‐like) under control and chilling conditions. Error bars represent SD values. The letters indicate significant differences according to one‐way ANOVA (Tukey's test; *P* < 0.05).

To evaluate the physiological function of *SlBBX19* under chilling stress, transgenic plants were subjected to chilling treatment. Prior to chilling stress, all genotypes exhibited healthy growth with no significant differences. However, following chilling treatment, *SlBBX19*‐OE plants displayed severe chilling injury symptoms, characterized by severe leaf wilting and mortality. In contrast, *SlBBX19*‐KO plants maintained a relatively healthy growth status, demonstrating significantly enhanced chilling tolerance compared to the wild type (WT) (Figure 4A). To investigate the role of *SlBBX19* in photosystem performance, chlorophyll fluorescence parameters were measured in WT, *SlBBX19*‐OE, and *SlBBX19*‐KO plants using an Imaging‐PAM system. Under normal growth conditions, the maximum quantum efficiency of PSII (Fv/Fm) remained comparable among genotypes, ranging from 0.76 to 0.78, indicating that PSII was not impaired (Figure 4B,C). However, the effective quantum yield of PSII (Y(II)) was markedly reduced in OE plants, whereas KO plants exhibited higher Y(II) values than WT plants (Figure 4D). Consistently, the electron transport rate (ETR) was significantly lower in OE lines but higher in KO lines compared with WT (Figure 4F). In contrast, the quantum yield of regulated non‐photochemical energy dissipation (Y(NPQ)) was substantially increased in OE plants and reduced in KO plants (Figure 4E). Following exposure to chilling stress, Fv/Fm, Y(II), and ETR decreased in all genotypes; however, the extent of reduction differed markedly among lines. OE plants exhibited the most severe decline in Fv/Fm, reaching values of 0.32–0.38, whereas WT and KO plants maintained higher values of 0.53 and 0.61–0.67, respectively (Figure 4B,C). Similarly, Y(II) and ETR were significantly lower in OE lines than in WT and KO plants, while KO plants consistently displayed the highest values (Figure 4D,F). These results indicate that overexpression of *SlBBX19* compromises PSII photochemical performance and electron transport capacity, whereas loss of gene function contributes to the maintenance of photosynthetic activity under chilling conditions. To investigate the physiological basis of these phenotypic differences, we assessed the accumulation of ROS. DAB (for H_2_O_2_) and NBT (for $O_{2}^{\cdot -}$) staining revealed that *SlBBX19*‐OE leaves accumulated significantly higher levels of ROS under chilling stress compared to WT, whereas *SlBBX19*‐KO leaves exhibited markedly reduced ROS accumulation (Figure 4G,H). Further physiological and biochemical analyses indicated that *SlBBX19*‐OE plants suffered more severe damage to their cellular membranes. Specifically, the relative electrical conductivity and malondialdehyde (MDA) content were significantly higher in *SlBBX19*‐OE plants than in WT and KO plants, indicating exacerbated lipid peroxidation (Figure 4I,J). Concurrently, the activities of key antioxidant enzymes, superoxide dismutase (SOD) and peroxidase (POD), were significantly lower in *SlBBX19*‐OE plants but significantly enhanced in *SlBBX19*‐KO plants (Figure 4K,L). These results suggest that *SlBBX19* overexpression compromises the antioxidant defense system, leading to impaired ROS scavenging and subsequent oxidative damage. We also examined the expression of cold‐responsive genes, upon chilling treatment, the transcript levels of *SlCBF1* and *SlCOR47like* were significantly upregulated in *SlBBX19*‐KO plants but strongly suppressed in *SlBBX19*‐OE plants, with WT plants showing intermediate levels (Figure 4N,O). Since the expression of the cold‐responsive genes *SlCBF1* and *SlCOR47like* was significantly altered in *SlBBX19* transgenic plants under chilling stress, we further investigated whether these genes are direct transcriptional targets of SlBBX19. Dual‐luciferase transient expression assays were performed using the promoters of *SlCBF1* and *SlCOR47like* fused to the LUC reporter. However, co‐expression of SlBBX19 did not significantly affect the promoter activities of either *SlCBF1* or *SlCOR47like* compared with the empty vector control (Figure S4). These results indicate that the suppression of cold‐responsive genes by SlBBX19 is mediated through an indirect regulatory mechanism. Collectively, these findings demonstrate that *SlBBX19* functions as a negative regulator of chilling tolerance in tomato.

### Transcriptomic analysis reveals that *SlBBX19* represses the expression of *SlLhc* family genes

**Figure 5:**
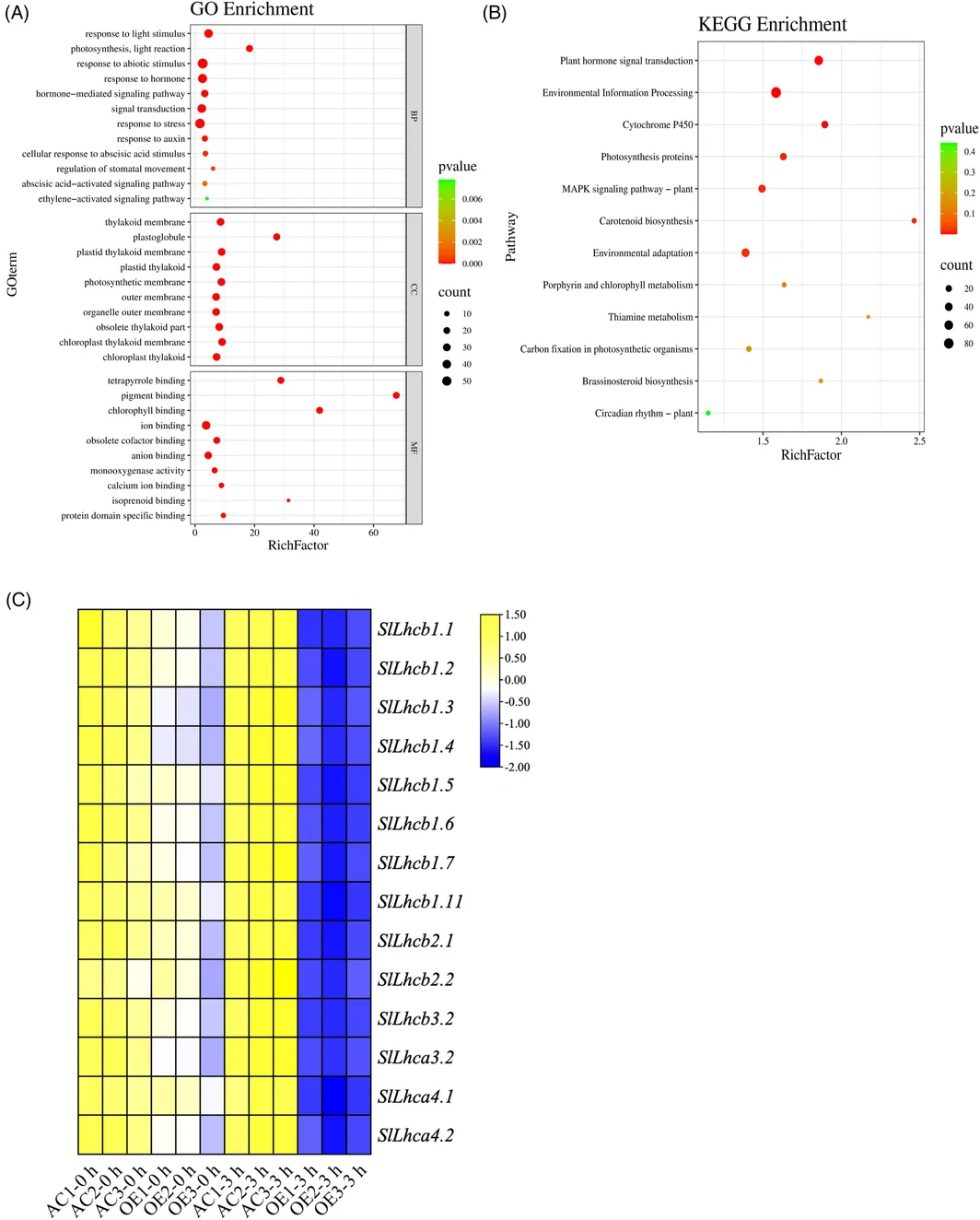
Transcriptomic analysis of the chilling stress response in *SlBBX19*‐OE and WT plants. (A) Gene Ontology (GO) enrichment analysis of differentially expressed genes (DEGs) in the *SlBBX19*‐OE plants at 3 h after chilling treatment. (B) Kyoto Encyclopedia of Genes and Genomes (KEGG) pathway enrichment analysis of DEGs in the *SlBBX19*‐OE plants at 3 h after chilling treatment. (C) Heatmap displaying the expression dynamics of *SlLhc* family genes in WT and *SlBBX19*‐OE plants at 0 and 3 h of chilling treatment.

To elucidate the molecular mechanism underlying *SlBBX19*‐mediated chilling tolerance, we performed RNA sequencing on *SlBBX19*‐OE and wild type (WT) plants subjected to chilling treatment (4°C) for 3 h. Gene ontology (GO) enrichment analysis revealed that differentially expressed genes (DEGs) were significantly enriched in biological processes such as photosynthesis, light reaction, response to abiotic stimulus, response to hormone, pigment binding, and chlorophyll binding (Figure 5A). KEGG pathway analysis further confirmed that these genes are primarily involved in plant hormone signal transduction, cytochrome P450, photosynthesis proteins, and carotenoid biosynthesis pathways (Figure 5B). These results suggest that *SlBBX19* likely functions by broadly regulating genes associated with photosynthesis and stress responses. To further validate these findings, we specifically examined the expression profiles of the *SlLhc* family genes encoding photosynthetic antenna proteins. Heatmap analysis showed that under normal growth conditions, the transcript levels of several *Lhc* family members (e.g., *SlLhcb1.1*, *SlLhcb1.3*, *SlLhcb1.4*, *SlLhcb1.5*, *SlLhcb2.2*, *SlLhcb3.2*, *SlLhca3.2*, *SlLhca4.1*, *SlLhca4.2*) were lower in *SlBBX19*‐OE plants compared to WT (Figure 5C). Notably, after 3 h of chilling treatment, the expression of the majority of *SlLhc* family genes was strongly repressed in *SlBBX19*‐OE plants, exhibiting lower levels than in WT (Figure 5C). Collectively, the transcriptomic data demonstrate that *SlBBX19* significantly suppresses the expression of *SlLhc* family genes under both normal and chilling stress conditions. This provides comprehensive transcriptomic evidence supporting the hypothesis that *SlBBX19* reduces photosynthetic efficiency by downregulating *SlLhc* family genes.

### SlBBX19 directly binds to the promoters of *SlLhcb2.2* and *SlLhca4.1* and negatively regulates their expression

**Figure 6:**
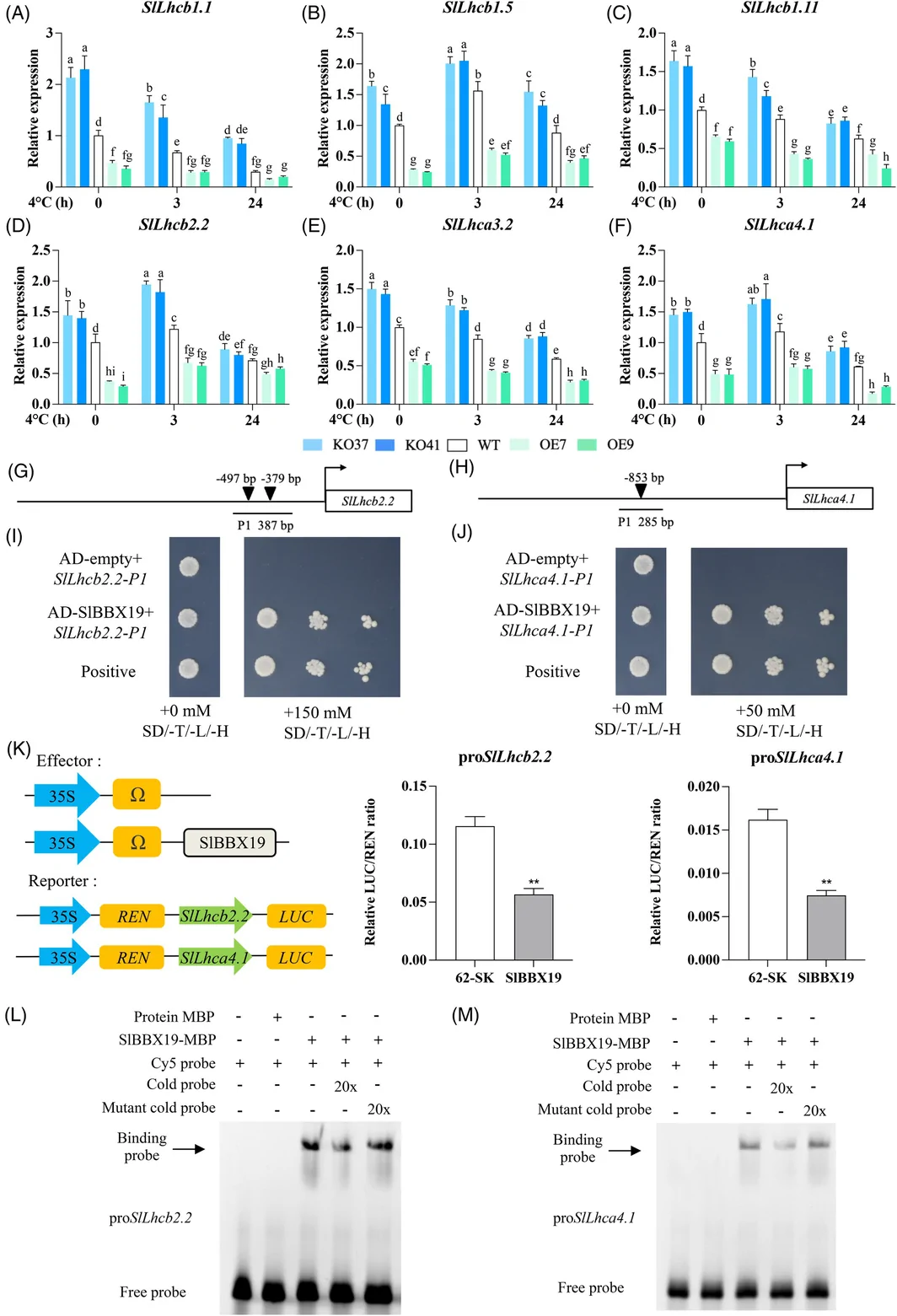
SlBBX19 directly binds to the promoters of *SlLhcb2.2* and *SlLhca4.1* and negatively regulates their expression. (A–F) Expression levels of six *SlLhcb* family genes in WT, *SlBBX19*‐KO, and *SlBBX19*‐OE plants under chilling stress. Error bars represent SD values. The letters indicate significant differences according to one‐way ANOVA (Tukey's test; *P* < 0.05). (G, H) Schematic representation of G‐box cis‐regulatory elements within the promoters of *SlLhcb2.2* and *SlLhca4.1*. (I, J) Yeast one‐hybrid (Y1H) assays verifying the direct interaction between SlBBX19 and the promoters of *SlLhcb2.2* and *SlLhca4.1*. Positive control: P53‐pHIS+pGADT7‐Rec53. Negative controls: *SlLhcb2.2*‐P1 or *SlLhca4.1*‐P1 co‐transformed with empty pGADT7 vector. (K) Dual‐luciferase reporter assays. Left: schematic of effector and reporter constructs. Right: SlBBX19 represses the transcriptional activity of the *SlLhcb2.2* and *SlLhca4.1* promoters. Error bars represent SD values. Statistical analysis was performed by two‐tailed Student's *t*‐test (**P < 0.01). (L, M) Electrophoretic mobility shift assays (EMSAs) demonstrating direct binding of SlBBX19 to promoter fragments of *SlLhcb2.2* and *SlLhca4.1*. The mutant probes contained an AAAAAA substitution at the target binding site. Bound DNA‐protein complexes are indicated by arrows.

To further validate the RNA‐seq results and identify the downstream targets of SlBBX19, we quantified the expression levels of *SlLhc* family members (*SlLhcb1.1*, *SlLhcb1.5*, *SlLhcb1.11*, *SlLhcb2.2*, *SlLhca3.2*, and *SlLhca4.1*) in WT, *SlBBX19*‐OE, and *SlBBX19*‐KO plants using qRT‐PCR. Under normal growth conditions, the transcript levels of these genes were significantly higher in *SlBBX19*‐KO plants than in WT, whereas they were lowest in *SlBBX19*‐OE plants (Figure 6A–F). Following chilling treatment, this expression pattern remained consistent: expression was highest in *SlBBX19*‐KO plants but strongly suppressed in *SlBBX19*‐OE plants (Figure 6A–F). These results further confirm the negative regulatory role of SlBBX19 on *SlLhc* family genes. To investigate whether SlBBX19 directly regulates these genes, we analyzed the promoter sequences of *SlLhcb2.2* and *SlLhca4.1* and identified the presence of G‐box elements, which are recognized by plant BBX transcription factors. Yeast One‐Hybrid (Y1H) assays demonstrated that SlBBX19 protein could specifically bind to the promoter fragments of *SlLhcb2.2* and *SlLhca4.1* (Figure 6I,J). Furthermore, Dual‐Luciferase (Dual‐LUC) reporter assays revealed that co‐expression of SlBBX19 significantly reduced the luciferase activity driven by the *SlLhcb2.2* and *SlLhca4.1* promoters, confirming the transcriptional repression activity of SlBBX19 (Figure 6K). To verify the direct binding and specificity of SlBBX19 to the promoters, we performed electrophoretic mobility shift assays (EMSA). The results showed that purified SlBBX19 protein formed specific complexes with the *SlLhcb2.2* and *SlLhca4.1* promoter probes containing G‐box elements (Figure 6L,M). This shifted band was significantly diminished by the addition of excess unlabeled competitor probes but was not affected by mutated competitor probes. This indicates that the binding of SlBBX19 to the promoter probes is specific to the G‐box motif. Collectively, these findings demonstrate that SlBBX19 directly binds to the G‐box elements within the *SlLhcb2.2* and *SlLhca4.1* promoters and inhibits their transcriptional activity. Given that BBX proteins often function together with cofactors such as HY5 or COP1‐associated components, we examined whether SlBBX19 interacts with SlHY5, SlCOP1, or SlBBX31. However, neither yeast two‐hybrid nor BiFC assays detected interactions between SlBBX19 and these proteins (Figure S5). However, we cannot exclude the possibility that SlBBX19 interacts with other regulatory proteins. Identification of these potential interacting partners will be an important direction for future studies.

### Silencing *SlLhcb2.2* or *SlLhca4.1* via VIGS inhibits photosynthesis

**Figure 7:**
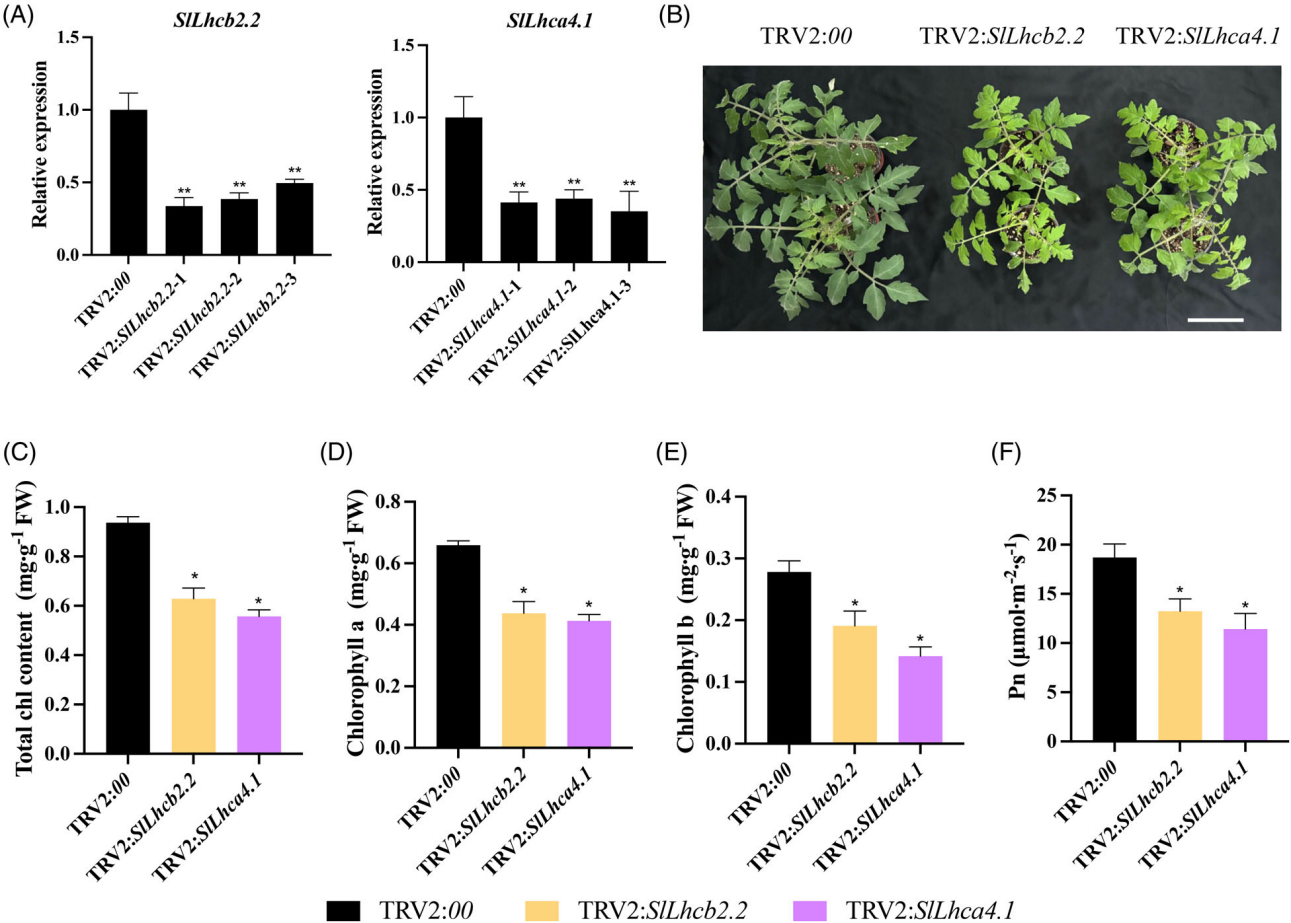
Silencing of *SlLhcb2.2* or *SlLhca4.1* compromises photosynthesis in tomato. (A) Verification of silencing efficiency in TRV2:*SlLhcb2.2* or TRV2:*SlLhca4.1* plants by qRT‐PCR. Error bars represent SD values. Statistical analysis was performed by two‐tailed Student's *t*‐test (**P < 0.01). (B) Representative photographs of 30‐day‐old TRV2:*SlLhcb2.2*, TRV2:*SlLhca4.1*, and control plants TRV2:*00*. Scale bar = 10 cm. (C–E) Chlorophyll content in leaves: (C) total chlorophyll, (D) chlorophyll a, and (E) chlorophyll b. (F) Net photosynthetic rate (Pn). Error bars represent SD values. Statistical analysis was performed by two‐tailed Student's *t*‐test (*P < 0.05).

To further validate the biological function of *SlLhcb2.2* and *SlLhca4.1* in photosynthesis and confirm whether they mediate the regulatory effects of *SlBBX19*, we employed virus‐induced gene silencing (VIGS) to mediate the silencing of these genes. First, we constructed TRV2 silencing vectors containing target gene fragments and inoculated tomato seedlings to generate silenced plants. Using *PHYTOENE DESATURASE* (*SlPDS*) as a visual marker for VIGS efficiency, we waited until TRV2:*SlPDS* plants exhibited the characteristic photobleaching phenotype (Figure S6), indicating that the silencing system was active. Subsequently, we assessed the silencing efficiency in the generated TRV2:*SlLhcb2.2* or TRV2:*SlLhca4.1* plants. qRT‐PCR results showed that the transcript levels of *SlLhcb2.2* or *SlLhca4.1* were significantly reduced in the corresponding silenced plants compared to the empty vector control plants (TRV2:*00*), confirming successful gene silencing (Figure 7A). Phenotypic observations revealed that the silenced plants exhibited reduced vegetative vigor compared to TRV2:*00* control plants. Moreover, the leaves of silenced plants displayed a lighter green coloration, whereas the control plants maintained a dark green phenotype (Figure 7B). Physiological assays further confirmed that both chlorophyll content and net photosynthetic rate were significantly lower in TRV2:*SlLhcb2.2* or TRV2:*SlLhca4.1* plants than in the TRV2:*00* plants (Figure 7C–F). Collectively, silencing *SlLhcb2.2* or *SlLhca4.1* recapitulated the photosynthetic defect phenotype observed in *SlBBX19*‐overexpressing plants. This indicates that these two genes are essential for maintaining normal photosynthetic efficiency, and their downregulation is sufficient to inhibit photosynthesis.

### Silencing *SlLhcb2.2* or *SlLhca4.1* reduces chilling tolerance

**Figure 8:**
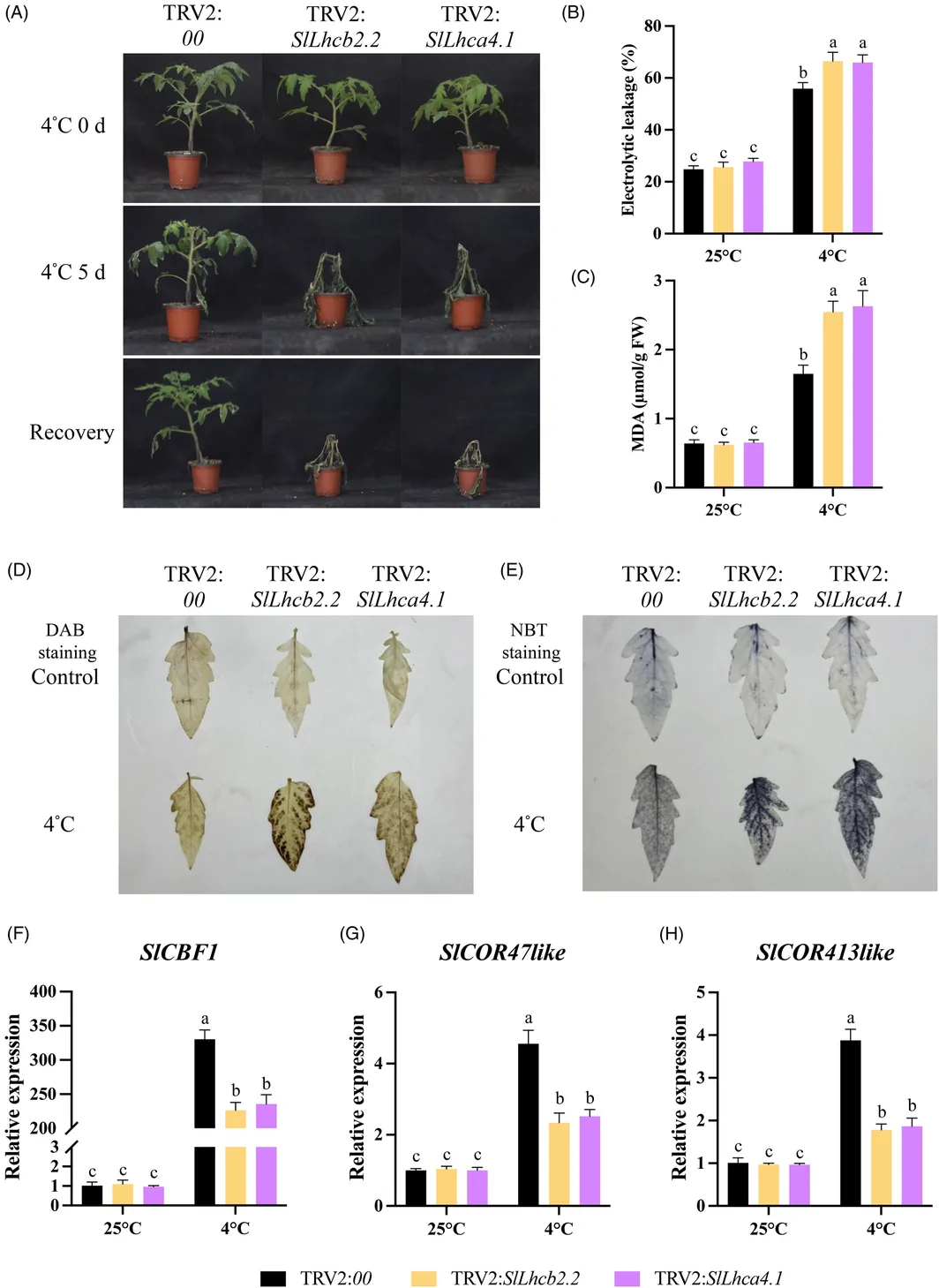
Silencing of *SlLhcb2.2* or *SlLhca4.1* attenuates chilling tolerance. (A) Representative phenotypes of TRV2:*SlLhcb2.2*, TRV2:*SlLhca4.1*, and TRV2 control plants after chilling treatment followed by 2 days of recovery under normal growth conditions. Ten plants per genotype were evaluated in each independent experiment, and three independent experiments were performed. (B) Relative electrolytic leakage under normal and chilling stress. (C) MDA content under normal and chilling stress. (D, E) Reactive oxygen species accumulation in leaves under control and chilling conditions: (D) DAB staining and (E) NBT staining. (F–H) Transcript levels of cold‐responsive genes (*SlCBF1*, *SlCOR47like*, and *SlCOR413like*) under control and chilling conditions. Error bars represent SD values. The letters indicate significant differences according to one‐way ANOVA (Tukey's test; *P* < 0.05).

To further investigate the biological function of *SlLhcb2.2* and *SlLhca4.1* under chilling stress, VIGS‐silenced plants (TRV2:*SlLhcb2.2* and TRV2:*SlLhca4.1*) were subjected to chilling treatment alongside the control plants (TRV2:*00*). Phenotypic observations revealed that silenced plants exhibited more severe chilling injury symptoms than control plants after 4°C treatment, indicating that silencing *SlLhcb2.2* or *SlLhca4.1* significantly compromised chilling tolerance (Figure 8A). Further physiological and biochemical assays elucidated the mechanism underlying this reduced tolerance. After 4°C treatment, the relative electrical conductivity and MDA content were higher in silenced plants than in TRV2:*00* plants, indicating more severe damage to the cellular membrane system (Figure 8B,C). Additionally, DAB and NBT staining showed that silenced plants accumulated higher levels of ROS under chilling stress, displaying more severe oxidative damage (Figure 8D,E). At the molecular level, we examined the expression of cold‐responsive genes. Under normal conditions, the expression of *SlCBF1*, *SlCOR47like*, and *SlCOR413like* showed no significant differences among genotypes. However, after 4°C treatment, the expression levels of these cold‐responsive genes were lower in silenced plants compared to control plants (Figure 8F–H). Collectively, *SlLhcb2.2* and *SlLhca4.1* function as positive regulators of chilling tolerance in tomato. They enhance plant adaptation to low temperatures by maintaining membrane integrity, scavenging ROS, and activating the expression of cold‐responsive genes. This finding also reciprocally confirms that *SlBBX19* compromises chilling tolerance by suppressing the expression of these two *SlLhc* genes.

## DISCUSSION

Overexpression of *SlBBX19* significantly affects tomato vegetative growth and reproductive development, including reduced plant height, lighter leaf color, decreased flower number, and accelerated fruit maturation. This pleiotropic phenotype is consistent with the general characteristic of BBX family members as integrators of environmental and endogenous developmental signals. For instance, regarding vegetative growth, overexpression of *SlBBX17* and *SlBBX31* leads to dwarfism, lighter green leaves, and smaller leaf size, accompanied by impaired photosynthesis (Wang & Zhan, 2024; Xu et al., 2022). In terms of reproductive development, *SlBBX28* promotes inflorescence branching and increases flower and fruit numbers (Lira et al., 2022); *CONSTANS‐like1* (*SlCOL1*) reduces flower and fruit numbers (Cui et al., 2022); *SlCOL4* integrates ABA and ethylene signals to inhibit fruit maturation (Wu, Tao, et al., 2025); and *SlBBX26* cooperates with *PHYTOCHROME‐INTERACTING FACTOR 4* (*SlPIF4*) to regulate GA and IAA homeostasis, thereby promoting fruit maturation (dos Reis Moreira et al., 2025). Suppressed growth coupled with accelerated fruit maturation typically reflects a reallocation of resources (trade‐off), which may facilitate the completion of the life cycle under adverse environmental conditions. Although the specific molecular mechanisms underlying *SlBBX19*‐mediated growth and maturation regulation were not further elucidated in this study, these results suggest that *SlBBX19* may coordinate growth development and environmental adaptation through light signaling or hormone‐related pathways, providing an important physiological context for its function under stress conditions.

The BBX transcription factor family is extensively involved in plant adaptation to abiotic stresses (Liu et al., 2022; Ma et al., 2024; Song et al., 2023; Wu et al., 2026), yet the functions of different members exhibit significant divergence under cold stress. Previous studies have shown that certain BBX proteins, such as *AtBBX7*, *AtBBX8*, *SlBBX17*, *SlBBX20*, *SlBBX31*, and *MdBBX37*, act as positive regulators of cold tolerance, enhancing plant cold resistance (An et al., 2021; Li et al., 2021; Ma, Chen, et al., 2025; Song et al., 2023; Zhu et al., 2023); whereas *AtBBX29* and *NtBBX9* have been reported as negative regulators, whose overexpression compromises cold stress tolerance (Liu et al., 2023; Wang, Shen, et al., 2023). In this study, we found that *SlBBX19* expression is significantly upregulated under cold stress, and the protein is localized to the nucleus with transcriptional regulatory activity. Although *SlBBX19* is cold‐induced, its overexpressing plants exhibit more severe oxidative damage and physiological disorders, with significantly reduced cold tolerance; conversely, cold tolerance is enhanced in knockout plants. Molecular analysis further revealed that under cold treatment conditions, the expression of canonical cold‐responsive genes *SlCBF1* and *SlCOR47like* was significantly suppressed in *SlBBX19*‐overexpressing plants. These results indicate that *SlBBX19* functions as a negative regulator of cold stress tolerance in tomato. Studies have shown that not all cold‐induced genes positively regulate chilling tolerance. Several TFs, including *AtMYB15* in Arabidopsis (Kim et al., 2017), *MdNAC029* in apple (An et al., 2018), *SlGATA22* in tomato (Wu, Zhao, et al., 2025), are induced by cold stress but function as negative regulators of chilling tolerance through distinct mechanisms. These studies suggest that cold‐induced negative regulators constitute an important regulatory strategy for preventing excessive or prolonged stress responses and maintaining cellular homeostasis. Similar to these regulators, *SlBBX19* is induced under chilling stress, yet genetically acts as a negative regulator of chilling tolerance. We therefore propose that SlBBX19 may function as a cold‐induced negative‐feedback regulator. Unlike the previously characterized tomato BBX proteins SlBBX17, SlBBX20, and SlBBX31, which positively regulate cold tolerance mainly through CBF signaling and ROS homeostasis, SlBBX19 functions as a negative regulator of chilling tolerance. More importantly, our data suggest that SlBBX19 directly represses *Lhc* genes, which are core components of the photosynthetic antenna system. These findings indicate that SlBBX19 may regulate cold adaptation through modulation of photosynthetic antenna components rather than solely through classical cold‐response pathways. Collectively, our findings establish a mechanistic link between photosynthetic antenna regulation and chilling tolerance mediated by SlBBX19, thereby expanding the functional diversity of BBX transcription factors in tomato cold responses.

Consistent with this distinct regulatory mechanism, transcriptomic analysis showed that *Lhc* related pathways were significantly enriched in *SlBBX19*‐mediated chilling responses, suggesting that regulation of the photosynthetic apparatus may be a key node for *SlBBX19* to coordinate stress adaptation. The traditional view holds that plants usually downregulate light‐harvesting complexes under low‐temperature stress to reduce excess excitation energy, thereby avoiding photoinhibition. However, this study found that transcriptional repression of specific *Lhc* members (*SlLhcb2.2* and *SlLhca4.1*) by *SlBBX19* instead exacerbated chilling injury. This indicates that in tomato, the functions of *SlLhcb2.2* and *SlLhca4.1* may go beyond simple light energy capture, and they may play an important role in maintaining the structural stability of Photosystem II (PSII) complexes under low temperature. Functional validation further confirmed the necessity of *SlLhcb2.2* and *SlLhca4.1* in chilling tolerance. The exacerbated oxidative damage and impaired cold‐responsive signaling caused by silencing these two genes were highly consistent with the phenotype of *SlBBX19* overexpression, indicating that the regulation of cold sensitivity by *SlBBX19* is largely achieved through the suppression of these two key targets. Recent studies have gradually revealed that Lhc proteins are not only structural components of photosynthesis but also participate in stress signal transduction and ROS scavenging networks. For example, silencing *SlLhcb1.11* or *EARLY LIGHTINDUCIBLE PROTEINS1* (*SlELIP1*) in tomato led to reduced cold tolerance in silenced plants (Meng et al., 2025). Overexpression of rapeseed *BnLhcb3.4* in Arabidopsis enhanced cold tolerance in transgenic plants (Zhang et al., 2022). Apple *MdLhcb4.3* enhanced tolerance to drought stress (Zhao et al., 2020), and wheat *TaLHC86* heterologously overexpressed in Arabidopsis enhanced salt tolerance (Chen et al., 2023). Our data support this view, that maintaining moderate expression levels of *SlLhcb2.2* and *SlLhca4.1* is crucial for the maintenance of cellular redox homeostasis under chilling stress. Therefore, as a negative regulator, excessive activation of *SlBBX19* may break the delicate balance between light energy absorption and photosystem stability, leading to electron transport chain blockage and ROS burst. This mechanism reveals a new pathway by which light signaling transcription factors integrate environmental stress signals through direct regulation of the photosynthetic apparatus, providing a new molecular perspective for understanding how photosynthetic antenna regulation contributes to chilling adaptation.

**Figure 9:**
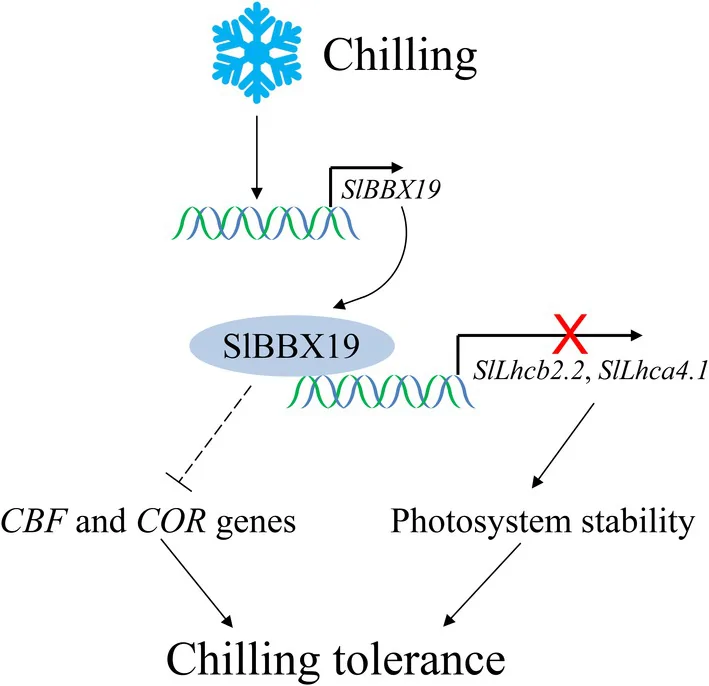
Proposed working model for *SlBBX19*‐mediated regulation of chilling tolerance in tomato. Under chilling stress, the expression of *SlBBX19* is transcriptionally upregulated. On one hand, SlBBX19 directly binds to the promoters of *SlLhcb2.2* and *SlLhca4.1* to repress their transcription. As positive regulators of chilling tolerance, *SlLhcb2.2* and *SlLhca4.1* are essential for maintaining photosystem stability; thus, their suppression compromises chilling tolerance. On the other hand, SlBBX19 represses the transcription of downstream cold‐responsive genes, including *CBF* and *COR* genes. Through these mechanisms, *SlBBX19* functions as a negative regulator that ultimately attenuates chilling tolerance in tomato plants.

Integrating the genetic, physiological, and molecular evidence from this study, we propose a working model for *SlBBX19*‐mediated chilling stress response in tomato (Figure 9). Under chilling stress, *SlBBX19* expression is induced, and its protein localizes to the nucleus to exert transcriptional regulatory activity. On one hand, SlBBX19 inhibits the expression of canonical cold‐responsive genes *CBF* and *COR* in the cold signaling pathway, limiting the activation of cold defense responses; on the other hand, SlBBX19 directly binds to and inhibits the transcription of *Lhc* genes *SlLhcb2.2* and *SlLhca4.1*, thereby compromising photosystem stability. Under chilling stress, an imbalance between light energy absorption and assimilation processes readily leads to the accumulation of excess excitation energy and ROS; therefore, Lhc‐mediated photosystem stability is crucial for maintaining cellular redox homeostasis and chilling tolerance. By simultaneously acting on the cold‐responsive signaling pathway and photosystem function, SlBBX19, as a negative regulator, integrates chilling stress signals with photosystem regulation, ultimately reducing tomato chilling stress tolerance.

## MATERIALS AND METHODS

### Plant materials and growth conditions

The tomato cultivar “*Alisa Craig*” (AC) was used as the genetic background for transformation. To construct the overexpression (OE) vector, the coding sequence (CDS) of *SlBBX19* was cloned into the pHellsgate2 vector driven by the *CaMV35S* promoter. The knockout (KO) vector harboring dual‐target sgRNAs was constructed using the pKSE402 vector. Tomato genetic transformation was performed following the previously described protocol (van Eck et al., 2019). The methods for assessing the response of *SlBBX19* to abiotic stresses and hormone treatments were conducted as previously reported (Liang et al., 2022).

Tomato plants were grown under normal conditions with a 16‐h light (25°C)/8‐h dark (22°C) photoperiod. When seedlings developed five true leaves, they were transferred to a growth chamber for chilling stress treatment. For the chilling assay of *SlBBX19* overexpression, knockout, and WT plants, seedlings were exposed to 4°C under a 16‐h light (10 000 lux)/8‐h dark photoperiod for 7 days. For the virus‐induced gene silencing (VIGS) plants, seedlings were subjected to 4°C under a 16‐h light (10 000 lux)/8‐h dark photoperiod for 5 days. After chilling treatment, all plants were returned to normal growth conditions for 2 days before survival was evaluated. Plants with a healthy shoot apical meristem after the recovery period were scored as survivors.

### RNA extraction and real‐time quantitative PCR

Samples were collected from tomato cultivar AC, including roots, stems, young leaves, mature leaves, flowers, fruits at different developmental stages (green ripe stage, breaker stage, and post‐breaker stage), and seeds. In addition, tomato leaf tissues subjected to various treatments, including 4°C, 42°C, polyethylene glycol (PEG), gibberellic acid (GA), and indole‐3‐acetic acid (IAA), were also harvested. Total RNA was extracted using TRIzol reagent, followed by first‐strand cDNA synthesis using the All‐in‐One RT MasterMix (Life‐iLab, China). Real‐time quantitative PCR (RT‐qPCR) was performed using NovoStart Fast SYBR qPCR SuperMix (Novoprotein, China) according to the manufacturer's instructions. *SlActin7* was used as the internal reference gene. The primers used for qPCR are listed in Table S1.

### Protein extraction and immunoblot analysis

The CDS of *SlBBX19* without the stop codon was fused to the pCAMBIA1300‐mCherry vector. *N. benthamiana* leaves transiently expressing SlBBX19‐mCherry were harvested at the indicated time points (0, 3, 6, and 12 h) after chilling treatment. Total proteins were extracted using RIPA extraction buffer (Solarbio, China). Equal amounts of total protein were separated by SDS‐PAGE and transferred onto PVDF membranes. The membranes were incubated with an anti‐mCherry antibody (ThermoFisher, USA). Protein signals were visualized using an enhanced chemiluminescence (ECL) detection system. An anti‐Actin antibody (ABclonal, China) was used to detect Actin as the loading control.

### Yeast transactivation assay

For the yeast auto‐activation assay, the coding sequence (CDS) of *SlBBX19* was cloned into the pGBKT7 vector using the Single EasyClone Kit (YoungGen, China). PCR verification was performed using 2× Taq Master Mix (GenScript, China), and positive clones were confirmed by sequencing prior to plasmid extraction. The confirmed recombinant plasmid was transformed into the yeast strain Y2HGold. Transformants were plated on SD/‐Trp and SD/‐Trp/‐His/‐Ade selective media and incubated at 28°C for 3–5 days to assess auto‐activation activity.

### Subcellular localization

For subcellular localization analysis, the CDS of *SlBBX19* without the stop codon was fused to the pBI121‐GFP vector. The recombinant construct was introduced into *Agrobacterium* strain GV3101 and subsequently infiltrated into tobacco leaves. After incubation in darkness for 2 days, GFP fluorescence signals were observed using a fluorescence microscope.

### Measurement of plant growth and fruit ripening correlation indices

To determine chlorophyll content, approximately 0.1 g of fresh tomato leaves was collected and placed in a 2 ml microcentrifuge tube. Samples were rapidly frozen in liquid nitrogen and ground using a tissue grinder. Subsequently, 1.5 ml of 80% (v/v) acetone was added, and the samples were incubated in darkness until the tissues were completely decolorized. The absorbance of the extracts was measured at 665, 649, and 470 nm using a microplate reader. Chlorophyll content was calculated according to previously reported methods (Chazaux et al., 2022). The net photosynthetic rate (Pn) and transpiration rate (Tr) were measured using a LI‐6800 portable photosynthesis system (LI‐COR, USA).

For fruit firmness measurement, fruits of *SlBBX19*‐OE, *SlBBX19*‐KO, and wild type (WT) plants were harvested at 35 and 38 days post anthesis (Dpa). Fruit firmness was determined using a hardness tester (HPE II Fff, Bareiss, Germany). Ethylene production and total carotenoid content were measured according to previously described methods (Liang et al., 2025).

### Measurement of chilling stress physiological indices

Electrolyte leakage, malondialdehyde (MDA) content, and the activities of superoxide dismutase (SOD) and peroxidase (POD) were measured according to previously described methods (Ma, Liang, et al., 2025; Wang, Hu, et al., 2023). Chlorophyll fluorescence parameters were determined using an IMAGING‐PAM chlorophyll fluorometer (Heinz Walz, Germany) following the manufacturer's instructions. Before measurements, leaves were dark‐adapted for 30 min. For NBT staining, leaves were vacuum‐infiltrated with DAB solution (1 mg ml^−1^ in 10 mM Na_2_HPO_4_, pH 4.0) for 5–10 min and then kept in the dark at room temperature for 10 h to visualize H_2_O_2_ accumulation. After washing, the leaves were decolorized using 75% ethanol until a clear background was achieved. Similarly, for NBT staining, leaves were infiltrated with a solution of 0.5 mg ml^−1^ NBT in 10 mM PBS (pH 7.8). The incubation was performed in darkness at room temperature for 3 h until blue‐black deposits appeared. Post‐incubation, samples were rinsed with water and bleached with 75% ethanol to remove chlorophyll interference.

### RNA sequencing (RNA‐seq)

For RNA‐seq analysis, *SlBBX19*‐OE and wild type (WT) tomato plants at the five‐true‐leaf stage were used. One group of plants was subjected to low‐temperature treatment at 4°C, while another group was maintained at 25°C as the control. Samples, including young leaves and shoot apices, were collected at 0 and 3 h after treatment and immediately frozen in liquid nitrogen. Three biological replicates were prepared for each time point. All samples were sent to Annoroad Gene Technology Co., Ltd. (Beijing, China) for sequencing. Raw sequencing data were processed using fastp for quality control to obtain clean reads. The clean reads were aligned to the tomato reference genome (SL4.0) using HISAT2. Gene expression levels were quantified using featureCounts, and differential expression analysis was performed using DESeq2 to identify differentially expressed genes (DEGs).

### Yeast one‐hybrid (Y1H)

Promoter fragments of *SlLhcb2.2* and *SlLhca4.1* containing the G‐box element were amplified and ligated into the pHis2 vector via homologous recombination. The CDS of *SlBBX19* was cloned into the pGADT7 vector. The pGADT7‐*SlBBX19* construct and the recombinant pHis2 plasmid were co‐transformed into the yeast strain Y187. Yeast one‐hybrid assays were performed according to the manufacturer's instructions. Transformants were plated on SD/‐Trp/‐Leu/‐His medium and incubated at 28°C for 5–7 days.

### Dual‐LUC assay

The promoter fragments of *SlCBF1*, *SlCOR47like*, *SlLhcb2.2*, and *SlLhca4.1* were individually cloned into the pGreenII 0800‐LUC vector, while the CDS of *SlBBX19* was inserted into the pGreenII 62‐SK vector. Verified constructs were transformed into *Agrobacterium* strain GV3101 harboring pSoup‐p19. Four‐week‐old tobacco leaves were infiltrated for transient expression analysis. After infiltration, plants were incubated at 25°C in darkness for 1 day, followed by 1 day under a normal light/dark cycle at 25°C. Luciferase activities were measured using a Dual‐Luciferase Reporter Gene Assay Kit (Coolaber, China), and the LUC/REN ratio was calculated.

### EMSA

The CDS of *SlBBX19* was cloned into the pMAL‐c5X vector and the recombinant plasmid was transformed into BL21 (DE3). The SlBBX19‐MBP fusion protein was purified using Dextrin Beads 6FF (Solarbio, China). EMSA was performed as previously described (Wang et al., 2018).

### Yeast two‐hybrid (Y2H) assay

The full‐length CDS of *SlBBX19* was inserted into the pGBKT7 vector as the bait construct, whereas the full‐length CDSs of *SlHY5*, *SlCOP1*, and *SlBBX31* were cloned into the pGADT7 vector as prey constructs. The bait and prey plasmids were co‐transformed into the yeast strain AH109. Transformants were selected on SD/‐Leu/‐Trp and SD/‐Leu/‐Trp/‐His/‐Ade medium to assess protein interactions.

### Bimolecular fluorescence complementation (BiFC) assay

To examine potential protein interactions, the CDS (without the stop codon) of *SlBBX19* was cloned into the pSPYCE vector to generate the SlBBX19‐cYFP fusion construct. The CDSs (without the stop codon) of *SlHY5*, *SlCOP1*, and *SlBBX31* were individually inserted into the pSPYNE vector to generate the corresponding nYFP fusion constructs. Recombinant plasmids were introduced into *Agrobacterium tumefaciens* strain GV3101 and co‐infiltrated into fully expanded leaves of *N. benthamiana*. Fluorescence signals were examined 48 h after infiltration using a BX53 fluorescence microscope (Olympus, Japan).

### Virus‐induced gene silencing (VIGS)

Gene‐specific silencing fragments of *SlLhcb2.2* and *SlLhca4.1* were designed using the SGN VIGS Tool (Fernandez‐Pozo et al., 2015). The amplified fragments were cloned into the pTRV2 vector to generate recombinant constructs, which were subsequently transformed into *Agrobacterium* strain GV3101. Tomato cultivar “*Alisa Craig*” (AC) was used as the host material for gene silencing. When seedlings developed the first true leaf, cotyledons were infiltrated with *Agrobacterium* cultures. Equal volumes of pTRV1 and different pTRV2 constructs (pTRV2:*00*, pTRV2:*SlLhcb2.2*, pTRV2:*SlLhca4.1*, and pTRV2:*SlPDS*) were mixed at a 1:1 ratio, and the final OD_600_ of the bacterial suspension was adjusted to 1.0 for infiltration.

### Statistical analysis

Statistical analysis was performed by two‐tailed Student's *t*‐test or one‐way analysis of variance and Tukey's multiple comparison tests.

## AUTHOR CONTRIBUTIONS

XZ, YL, and FM conceived the experiments. YL, FyM, and FM performed the experiments. YL wrote and revised the article. HyS and HwS analyzed the data.

## CONFLICT OF INTEREST

The authors declare no competing interests.

## Supporting information


**Figure S1.** Identification of the chilling‐responsive *SlBBX19* from *SlBBX* family genes.
**Figure S2.** Phylogenetic and domain architecture analysis of the tomato BBX protein family.
**Figure S3.** *SlBBX19* has no significant effect on single fruit weight or fruit size.
**Figure S4.** SlBBX19 does not significantly affect the promoter activities of *SlCBF1* and *SlCOR47like* in dual‐luciferase assays.
**Figure S5.** No interactions were detected between SlBBX19 and SlHY5, SlCOP1, or SlBBX31.
**Figure S6.** Virus‐induced gene silencing (VIGS)‐mediated silencing of *SlPDS* induces an albino phenotype and validates silencing efficiency.

**Table S1.** Primers used in this study.

**Table S2.** Normalized RNA‐seq expression values (TPM) of genes in *SlBBX19* transgenic lines under chilling stress.

**Table S3.** Differentially expressed genes (DEGs) identified from RNA‐seq analysis of *SlBBX19* transgenic lines under chilling stress.

## Data Availability

The data that support the results are provided in this paper and its Supplementary Files.
