# Supplementary material for: SlBBX19 modulates plant development and negatively regulates chilling tolerance in tomato
Source: Plant J. 2026 Sep 7;127(5):e71099. doi: 10.1111/tpj.71099 (PMC13548794; doi:10.1111/tpj.71099)
Supplement: Supplementary file 1 [file TPJ-127-0-s001.pdf]

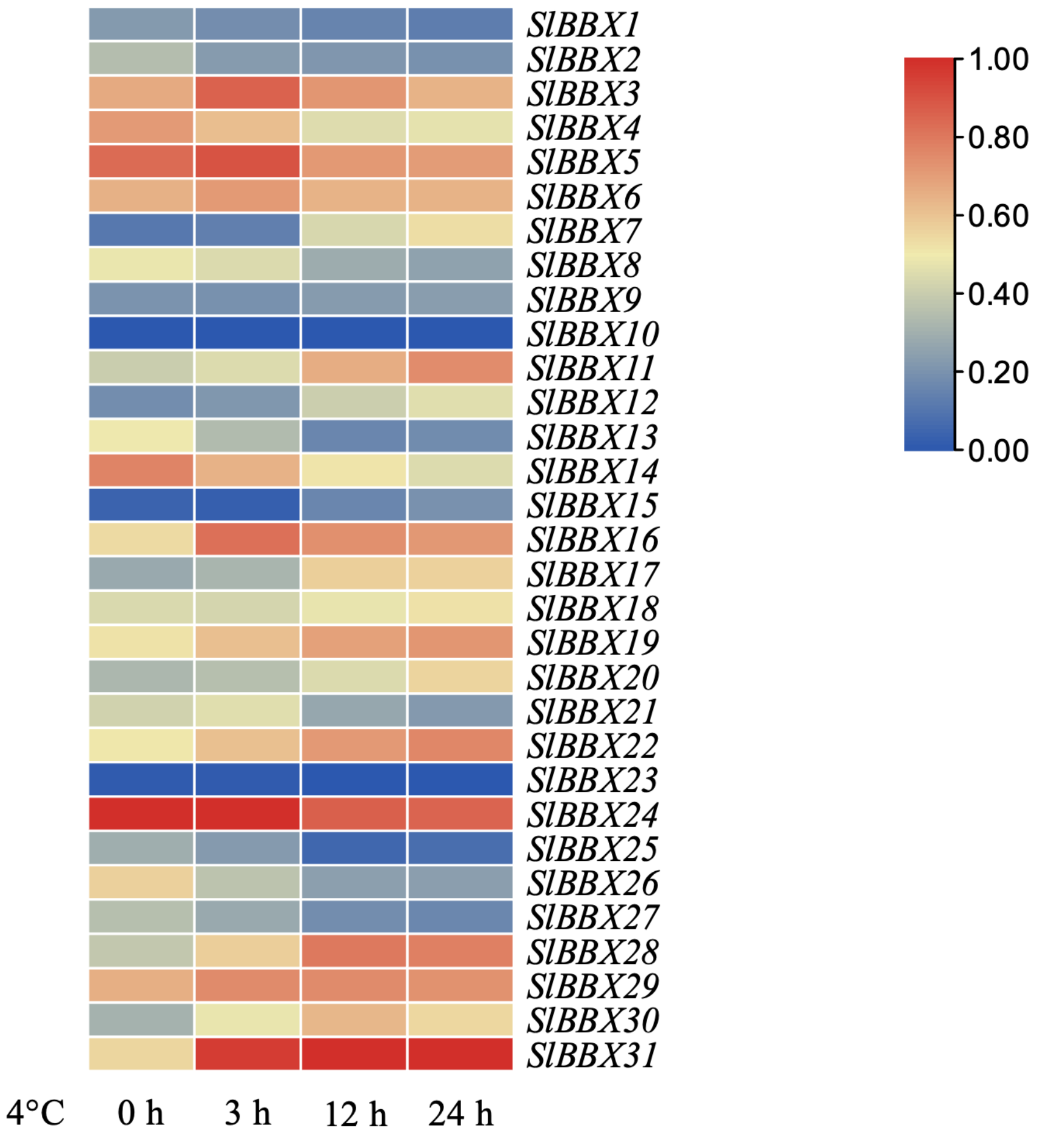

Figure S1. Identification of the chilling-responsive *SlBBX19* from *SlBBX* family genes.

RNA-seq data shown that *SlBBX19* is upregulated by chilling treatment in tomato. 4-week-old tomato 'AC' plants grown in soil were chilling treatment for 0, 3, 12, or 24 hours..

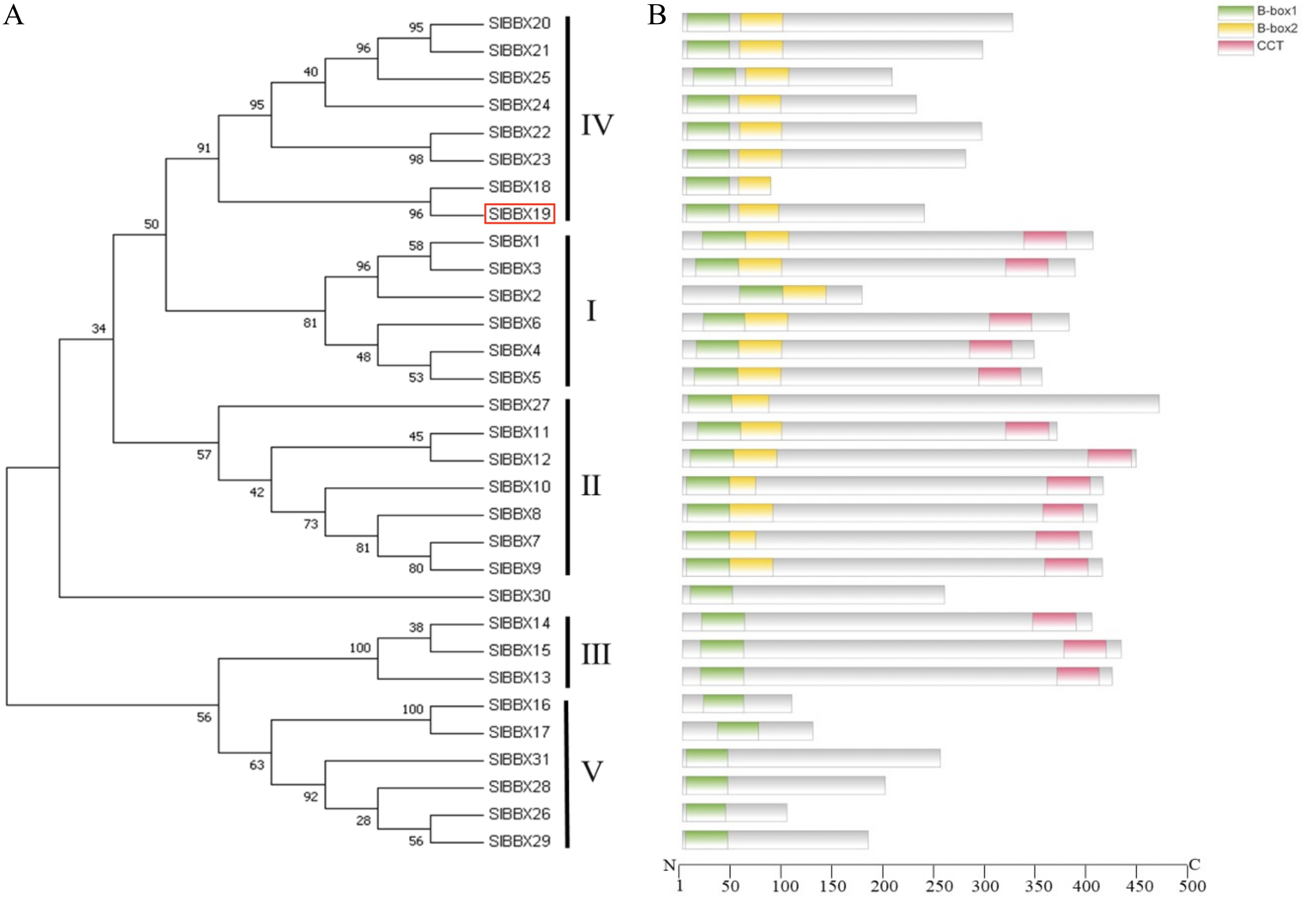

Figure S2. Phylogenetic and domain architecture analysis of the tomato BBX protein family.
(A) A total of 31 BBX family members were identified in the tomato genome and clustered into five subfamilies based on phylogenetic analysis. (B) Domain architecture analysis revealed that individual members contain either one or two B-box domains, with a subset additionally harboring a CCT domain. Phylogenetic tree was constructed using Neighbor-Joining method with 1000 bootstrap replicates. Protein sequences were retrieved from Solanum Genome Database.

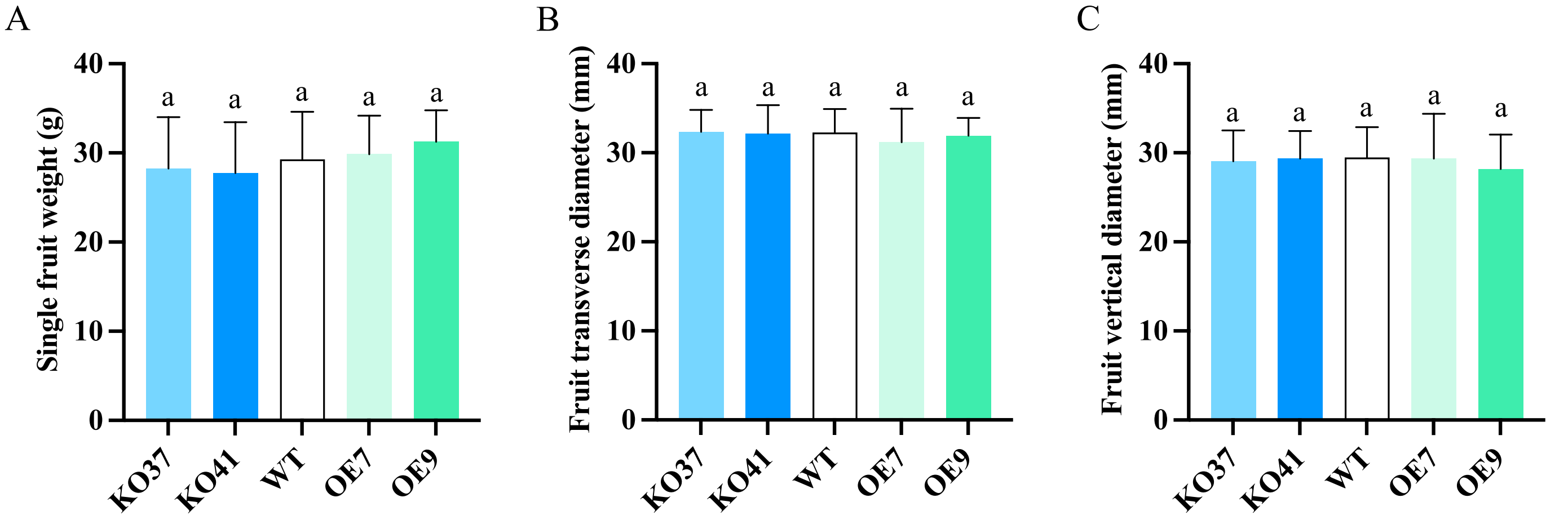

Figure S3. *SlBBX19* has no significant effect on single fruit weight or fruit size**.**
(A-C) Morphometric measurements of fruits from *SlBBX19*-OE, *SlBBX19*-KO, and WT plants: (A) single fruit weight, (B) fruit transverse diameter, and (C) fruit vertical diameter. Error bars represent SD values. The letters indicate significant differences according to one-way ANOVA (Tukey's test; $p < 0.05$).

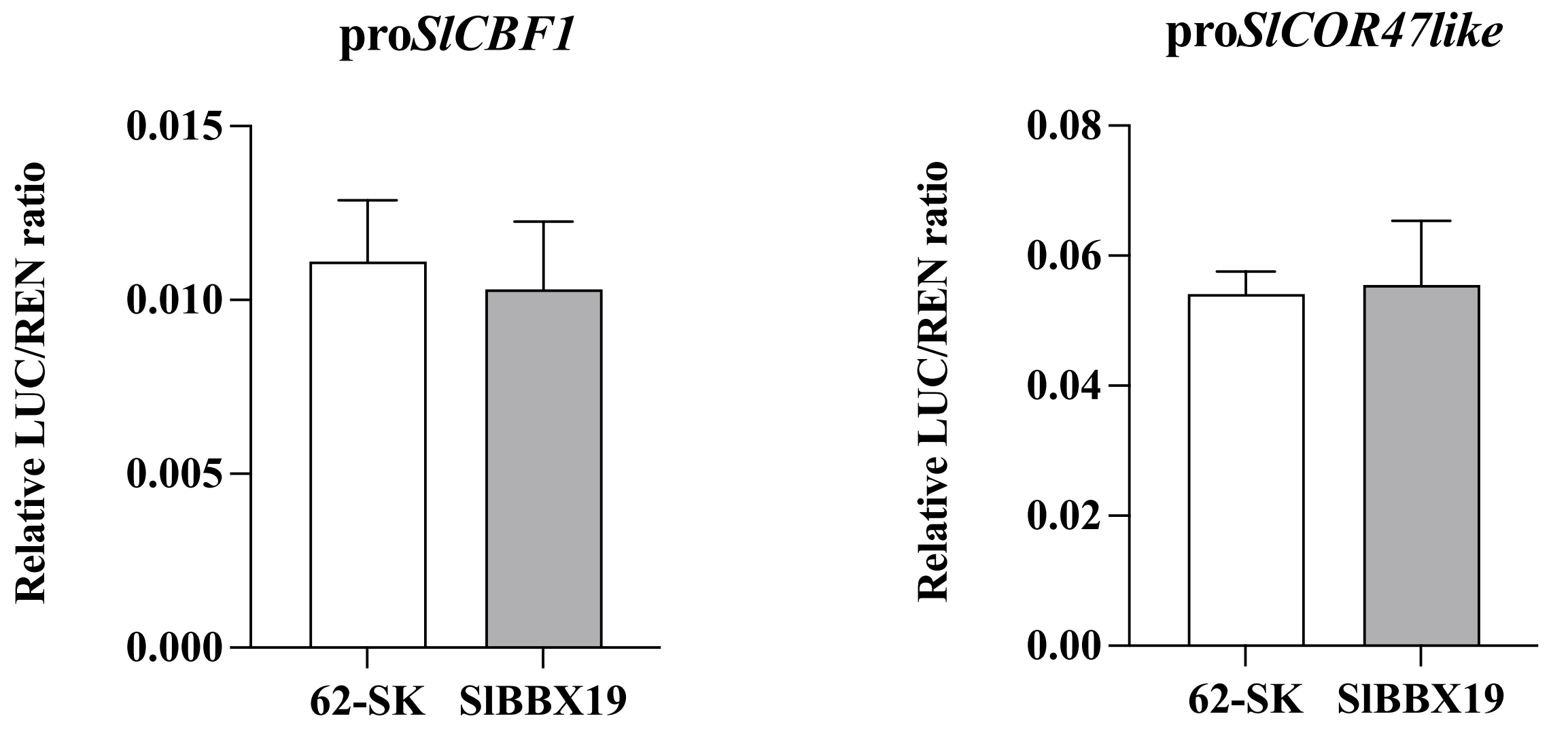

Figure S4. SlBBX19 does not significantly affect the promoter activities of *SlCBF1* and *SlCOR47like* in dual-luciferase assays. Error bars represent SD values. No significant differences were detected according to Student's *t*-test ($p > 0.05$).

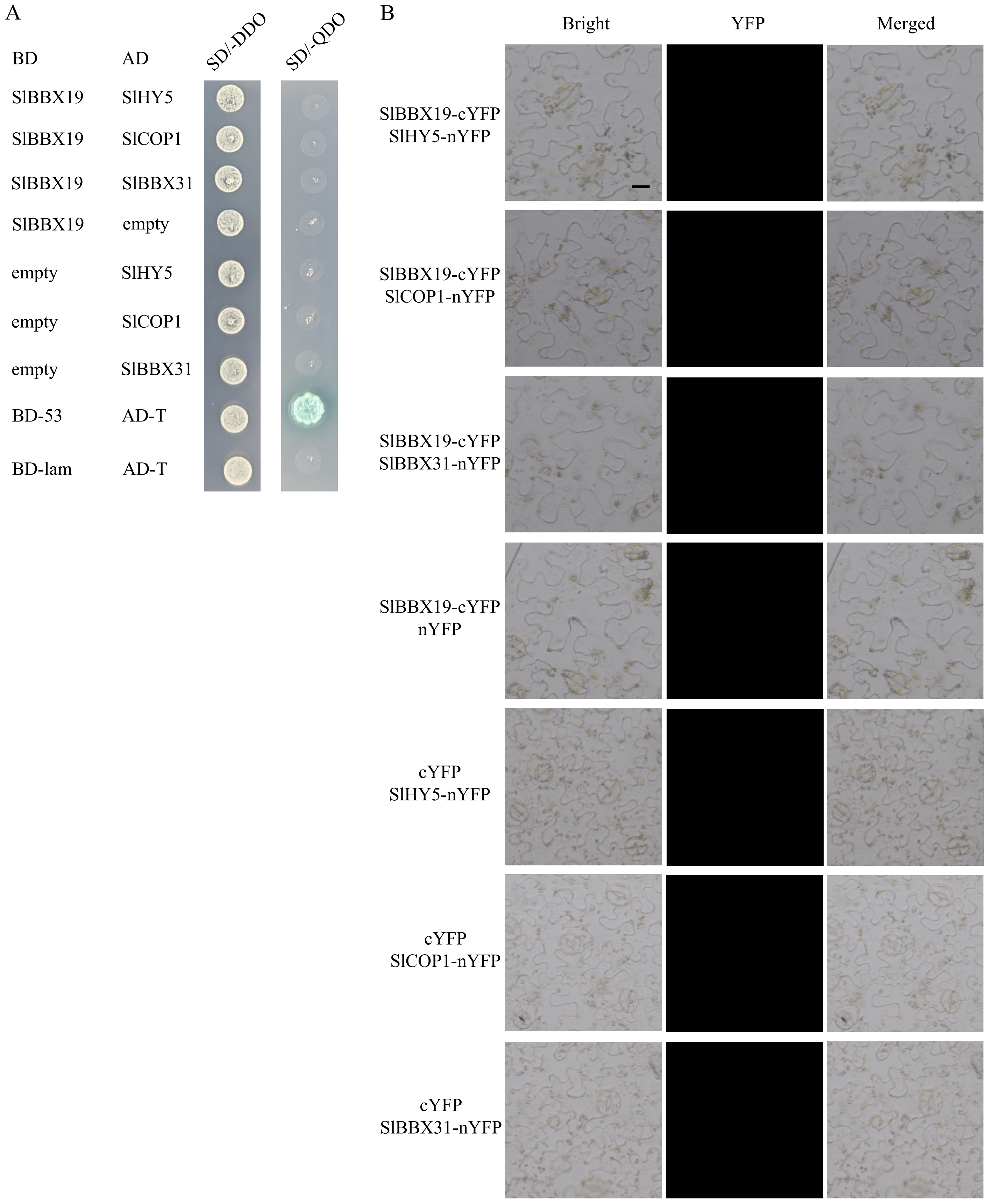

Figure S5. No interactions were detected between SlBBX19 and SlHY5, SlCOP1, or SlBBX31.
(A) Yeast two-hybrid (Y2H) assays examining the interactions between SlBBX19 and SlHY5, SlCOP1, or SlBBX31. Yeast cells were grown on SD/-Leu/-Trp (DDO) medium to verify co-transformation and subsequently transferred to SD/-Leu/-Trp/-His/-Ade (QDO) medium for interaction analysis. No protein-protein interactions were detected.
(B) Bimolecular fluorescence complementation (BiFC) assays in *Nicotiana benthamiana* leaves. No YFP fluorescence signal was observed in leaves co-expressing SlBBX19 with SlHY5, SlCOP1, or SlBBX31, indicating that no interaction was detected under the experimental conditions. Scale bar = 20 μm.

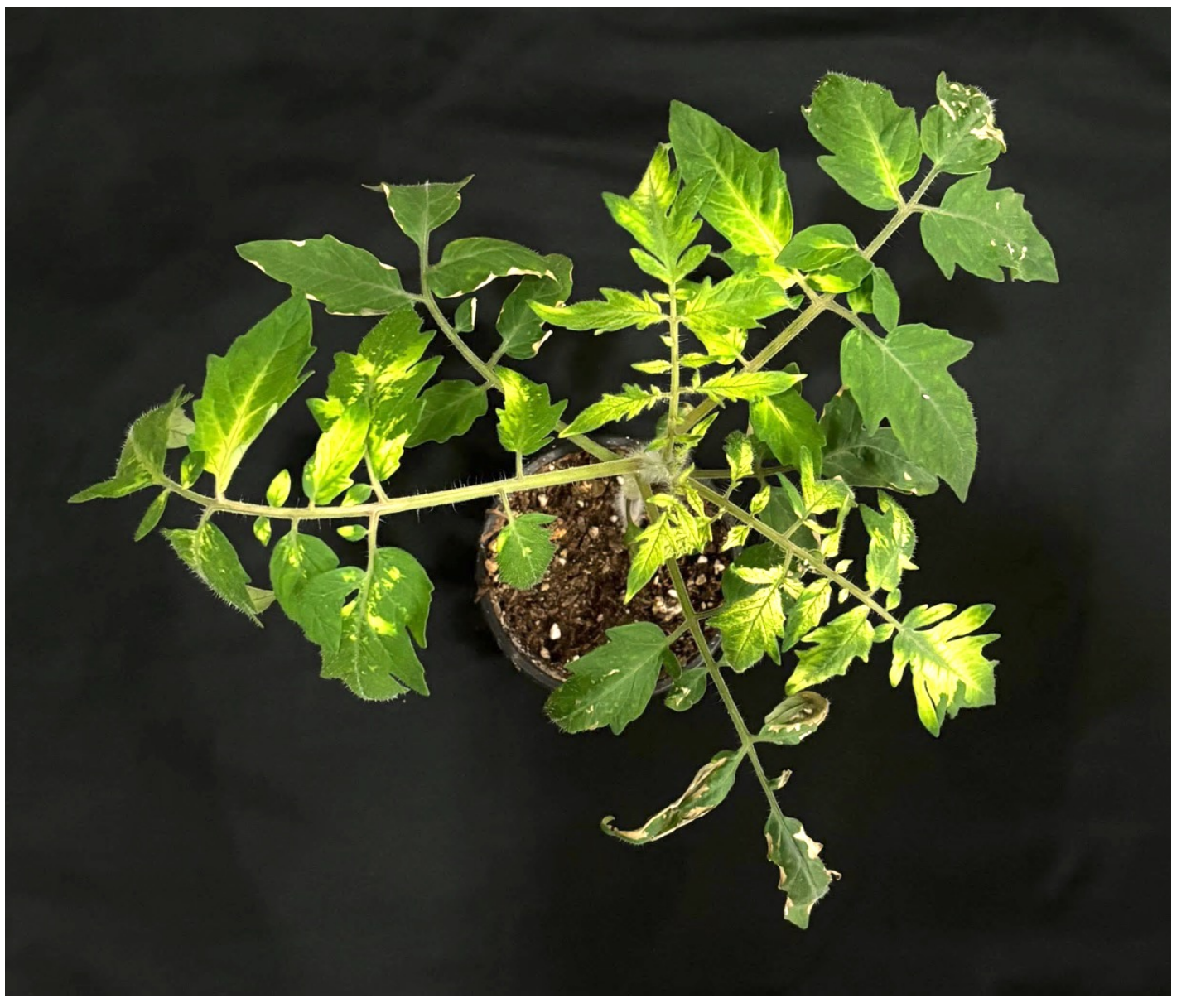

Figure S6. VIGS-mediated silencing of *SlPDS* induces an albino phenotype and validates silencing efficiency. Representative photographs of the albino phenotype in TRV2:*SlPDS* plants. The albinization resulting from *SlPDS* silencing serves as a reliable visual indicator for successful TRV2 delivery and silencing competence. Upon observation of a stable albino phenotype, confirming the functionality of the silencing system, the silencing efficiencies of the target genes *SlLhcb2.2* and *SlLhca4.1* were subsequently quantified.
